# Clinical efficacy of Osteoking in knee osteoarthritis therapy: a prospective, multicenter, non-randomized controlled study in China

**DOI:** 10.3389/fphar.2024.1381936

**Published:** 2024-06-28

**Authors:** Jun Zhou, Zelu Zheng, Yuxin Luo, Yawei Dong, Yan Yan, Yi Zhang, Kaiqiang Tang, Rui Quan, Jiaming Lin, Kuayue Zhang, Pengxuan Dong, Rongtian Wang, Haijun He, Na Lin, Xisheng Weng, Baohong Mi, Yanqiong Zhang, Weiheng Chen

**Affiliations:** ^1^ Department of Mini-Invasive Joint surgery, Beijing University of Chinese Medicine Third Affiliated Hospital, Beijing, China; ^2^ Engineering Research Center of Chinese Orthopaedic and Sports Rehabilitation Artificial Intelligent, Ministry of Education, Beijing, China; ^3^ Institute of Chinese Materia Medica, China Academy of Chinese Medical Sciences, Beijing, China; ^4^ Chinese Academy of Traditional Chinese Medicine, Wangjing Hospital, Beijing, China; ^5^ Department of Orthopedic Surgery, State Key Laboratory of Complex Severe and Rare Diseases, Peking Union Medical College Hospital, Chinese Academy of Medical Science and Peking Union Medical College, Beijing, China

**Keywords:** knee osteoarthritis, Osteoking, nonsteroidal anti-inflammatory drugs, clinical efficacy, safety

## Abstract

**Background:**

Osteoking has been extensively used for the treatment of knee osteoarthritis (KOA). However, it is lack of high-quality evidence on the clinical efficacy of Osteoking against KOA and the comparison with that of nonsteroidal anti-inflammatory drugs (NSAIDs).

**Aims:**

To evaluate the efficacy and safety of Osteoking in treating KOA.

**Methods:**

In the current study, a total of 501 subjects were recruited from 20 medical centers, and were divided into the Osteoking treatment group (*n* = 428) and the NSAIDs treatment group (*n* = 73). The Propensity Score Matching method was used to balance baseline data of different groups. Then, the therapeutic effects of Osteoking and NSAIDs against KOA were evaluated using VAS score, WOMAC score, EQ-5D-3L and EQ-VAS, while the safety of the two treatment were both assessed based on dry mouth, dizziness, diarrhea, etc.

**Results:**

After 8 weeks of treatment, the Osteoking group was compared with the NSAIDs group, the VAS score [2.00 (1.00, 3.00) vs. 3.00 (2.00, 4.00)], WOMAC pain score [10.00 (8.00, 13.00) vs. 11.00 (8.00, 16.00) ], WOMAC physical function score [32.00 (23.00, 39.00) vs. 39.07 ± 16.45], WOMAC total score [44.00 (31.00, 55.00) vs. 53.31 ± 22.47) ], EQ-5D-3L score [0.91 (0.73, 0.91) vs. 0.73 (0.63, 0.83) ] and EQ-VAS score [80.00 (79.00, 90.00) vs. 80.00 (70.00, 84.00) ] were improved by the treatment of Osteoking for 8 weeks more effectively than that by the treatment of NSAIDs. After 8 weeks of treatment with Osteoking, the VAS scores of KOA patients with the treatment of Osteoking for 8 weeks were reduced from 6.00 (5.00, 7.00) to 2.00 (1.00, 3.00) (*p* < 0.05), which was better than those with the treatment of NSAIDs starting from 2 weeks during this clinical observation. Importantly, further subgroup analysis revealed that the treatment of Osteoking was more suitable for alleviating various clinical symptoms of KOA patients over 65 years old, with female, KL II-III grade and VAS 4-7 scores, while the clinical efficacy of NSAIDs was better in KOA patients under 65 years old and with VAS 8–10 scores. Of note, there were no differences in adverse events and adverse reactions between the treatment groups of the two drugs.

**Conclusion:**

Osteoking may exert a satisfying efficacy in relieving joint pain and improving life quality of KOA patients without any adverse reactions, especially for patients with KL II-III grades and VAS 4–7 scores.

**Clinical Trial Registration:**

https://www.chictr.org.cn/showproj.html?proj=55387, Identifier ChiCTR2000034475

## 1 Introduction

Knee osteoarthritis (KOA) represents one of the most common musculoskeletal diseases and a major cause of disability in the elderly (Jang et al., 2021). With the acceleration of aging, the growth of the obese population and the extension of life expectancy, the prevention and treatment of KOA face severe challenges (Mahmoudian et al., 2021). Until 2020, the number of KOA patients has been increased to 317 million and is still growing worldwide. It is estimated that by 2050, the number of KOA patients will be reached 642 million (GBD, 2023; Mahmoudian et al., 2021). In China, the prevalence rate of symptomatic KOA among people over 65 years old is 60%, and the detection rate of radiological KOA is as high as 80% (Huang et al., 2018). The characteristics of KOA are long-term and irreversible diseases, which can cause pain, inconvenience, and even disability, placing a huge burden on patients’ physical and mental health and seriously interfering with their quality of life (Hattori et al., 2024). In addition, the high medical costs and corresponding indirect costs of KOA not only increase the economic burden on individuals and families, but also have a negative impact on the national medical system, and increase social and economic costs (Tang et al., 2016). Conservative treatment, as a first-line treatment to slow down disease progression, and to avoid or delay knee replacement surgery, has become increasingly important in the long-term management of KOA (Lim and Al-Dadah, 2022). Conservative treatment is mainly based on pain control and cartilage nutrition programs, with limited effects on the joints. In fact, KOA involves multiple lesions and complex pathological changes, which are the result of long-term interweaving of multiple pathogenic factors. The efficacy of local and single Western medicine treatment is limited (Savvari et al., 2023). In addition to the conventional treatment of KOA, traditional Chinese medicine is also an essential therapeutic strategy, which has been widely used in China and other Asian countries. According to statistics based on more than 30000 KOA patients in Taiwan, China, 76.7% of them have applied traditional Chinese medicine (including traditional Chinese patent medicines and simple preparations and acupuncture and moxibustion), and the final joint replacement rate is lower than that of western medicine (Lo et al., 2019).

Traditional Chinese Medicine (TCM) classifies KOA as “impediment disease.” According to TCM theory, KOA is a syndrome of deficiency of the root and excess of the branch. The invasion of external pathogens such as wind, cold, and dampness is the manifestation of the disease, while the deficiency of the liver and kidney is the root cause of the disease. The obstruction of the meridians is the central link in the pathogenesis. Osteoking originated from the Yi ethnic group in Yunnan Province, China, and is also known as Henggu Gushang Yuheji. Osteoking is a traditional Chinese patent medicines and simple preparations approved for marketing by the State Food and Drug Administration in 2002, and is a commonly used drug for KOA treatment. Osteoking is a commonly used medication for KOA therapy with a satisfying clinical efficacy (Li et al., 2023; Ling et al., 2021). Our preliminary study indicated that Osteoking may promote bone formation by regulating ZBP1–STAT1–PKR axis, leading to inhibit RIPK1/RIPK3/MLKL activation-mediated necroptosis (Zhang et al., 2024). However, it is lack of high-quality evidence on the clinical efficacy of Osteoking against KOA and the comparison with that of nonsteroidal anti-inflammatory drugs (NSAIDs). Therefore, we designed a prospective, multicenter, non-randomized controlled study to further evaluate the clinical efficacy and safety of Osteoking in the treatment of KOA from a macro perspective.

## 2 Material and methods

### 2.1 Investigational medications

As a “Type A extract” (Heinrich et al., 2022), Osteoking is composed of eight botanical drugs and one animal-derived drug. Utilizing modern pharmaceutical technology, these nine drugs were combined in various ratios, subsequently extracted, concentrated, and filtered to produce Osteoking. The proportion of drugs contained in each standard dose (1,000 mL) of Osteoking is as follows: Citrus reticulata Blanco [Rutaceae]: 10 g, Carthamus tinctorius L. [Asteraceae]: 15 g, Panax notoginseng (Burkill) F.H.Chen [Araliaceae]: 30 g, Eucommia ulmoides Oliv. [Eucommiaceae]: 30 g, Panax ginseng C.A.Mey. [Araliaceae]: 20 g, Astragalus mongholicus Bunge [Fabaceae]: 40 g, Datura metel L. [Solanaceae]: 6 g, Schizophragma integrifolium Oliv, a synonym of Hydrangea ampla (Chun) Y.De Smet & Granados [Hydrangeaceae]: 10 g, Carapax Trionycis, The back shell of the animal Trionyx sinensis Wiegmann of family Trionychidae: 10 g. Osteoking has been approved by the National Medical Products Administration (NMPA) (approval number: Z20025103). The oral liquid was manufactured by Yunnan Crystal Natural Pharmaceutical Co., Ltd. (Batch number: 20201016/202104026) (Supplementary Material S1–3).

### 2.2 Study design

We designed a nationwide, prospective, multi-center, non-randomized controlled study to evaluate the efficacy and safety of Osteoking in the treatment of KOA. From 1 May 2020 to 31 December 2021, patients with KOA were recruited from 12 provinces/municipalities directly under the central government, 20 medical centers, 13 western hospitals, and 7 traditional Chinese medicine hospitals in China. The detailed information of the clinical trial center and the proportion of patient sources are shown in Supplementary Material S4. The design and protocol of this study have been approved by the Ethics Review Committee of Peking Union Medical College Hospital (No. HS-2363) and the Medical Ethics Committee of Wangjing Hospital of Chinese Academy of Traditional Chinese Medicine (No. WJEC-KY-2020-006-P003). This study has been registered in the China Clinical Trial Registration Center (Registration No. ChiCTR2000034475).

### 2.3 Participants

All of patients who met the following characteristics were included (Kolasinski et al., 2020): 1) Subjects must meet the 1995 KOA diagnostic criteria of the American College of Rheumatology; 2) age ≥40 years; 3) signed informed consent, voluntary subjects; 4) good compliance with observation and evaluation, able to complete treatment as required.

Patients were excepted from this study if they met one or more: 1) Non-primary arthritis (traumatic arthritis, rheumatoid arthritis, gouty arthritis, metabolic bone disease, etc.); 2) Patients with severe medical or psychiatric conditions that prevent them from cooperating with the researchers; 3) Pregnant or lactating women; 4) Allergic individuals and those who are allergic to multiple drugs; 5) Use of other drug therapies in addition to the intervention method.

Patients withdrew from the study for any of the following reasons: 1) unwillingness to participate in the study; 2) injury, fracture, or specific pathological changes occurred during the trial, requiring the intervention to be stopped; 3) any allergic reaction or serious adverse event occurred.

### 2.4 Intervention

The experimental group was given Osteoking, 25 mL per oral administration, once every 2 days, for 12 days as a course of treatment, for a total of two courses. The control group was given non-steroidal anti-inflammatory drugs orally, including loxoprofen sodium tablets and celecoxib capsules. The specific course of treatment was recommended by international guidelines (Bannuru et al., 2019; Brophy and Fillingham, 2022).

### 2.5 Outcome

Patients were evaluated before treatment and at 2, 4, and 8 weeks of treatment.

The primary outcome measure was the VAS score. The primary end point is the VAS score after 8 weeks of treatment.

The second outcome measure include: 1) WOMAC total score and WOMAC subscale scores (WOMAC pain score, WOMAC stiffness score, and WOMAC physical function score). 2) The EuroQol Five Dimensions descriptice system (EQ-5D-3L) scale and the EuroQol Visual Analogue Scale (EQ-VAS) (EuroQol Group, 1990). Using the Chinese residents’ quality of life utility value scoring system (Luo et al., 2017), the specific calculation formula and evaluation system can be found in Supplementary Material S5.

### 2.6 Adverse events

Adverse events are defined as any adverse medical events related to the treatment regimen that result in persistent or worsening symptoms in patients requiring additional intervention. In this study, patients were asked to report any adverse outcomes they considered to be related to treatment, including complications, signs, or symptoms, at each follow-up visit. The incidence of adverse events is considered an indicator of safety evaluation.

### 2.7 Statistical analysis

Statistical analysis was performed using SPSS 26.0 software. Hypothesis testing was conducted using a two-sided test, with *p* < 0.05 indicating statistical significance. Descriptions of quantitative indicators were presented as N (%), Mean ± SD, or Median (Q1, Q3). Comparisons between two groups were measured using independent sample t-tests or Mann-Whitney U rank sum tests for continuous data, and chi-square tests or Fisher’s exact tests for discrete data. Generalized linear mixed models (GLMM) were used to compare repeated measurements between groups. The model included treatment, time, and the interaction between treatment and time as fixed effects, and patient-specific random intercepts. Patients were stratified by age, gender, KL grade, and Chinese Medicine staging (CMS) (Chen W. et al., 2023) (CMS I: VAS 0–3, CMS II: VAS 4–7, CMS III: VAS 8–10). GLMM were used to compare the efficacy of different populations in the two groups.

## 3 Results

### 3.1 Demographics and baseline characteristics of the patient

**FIGURE 1:**
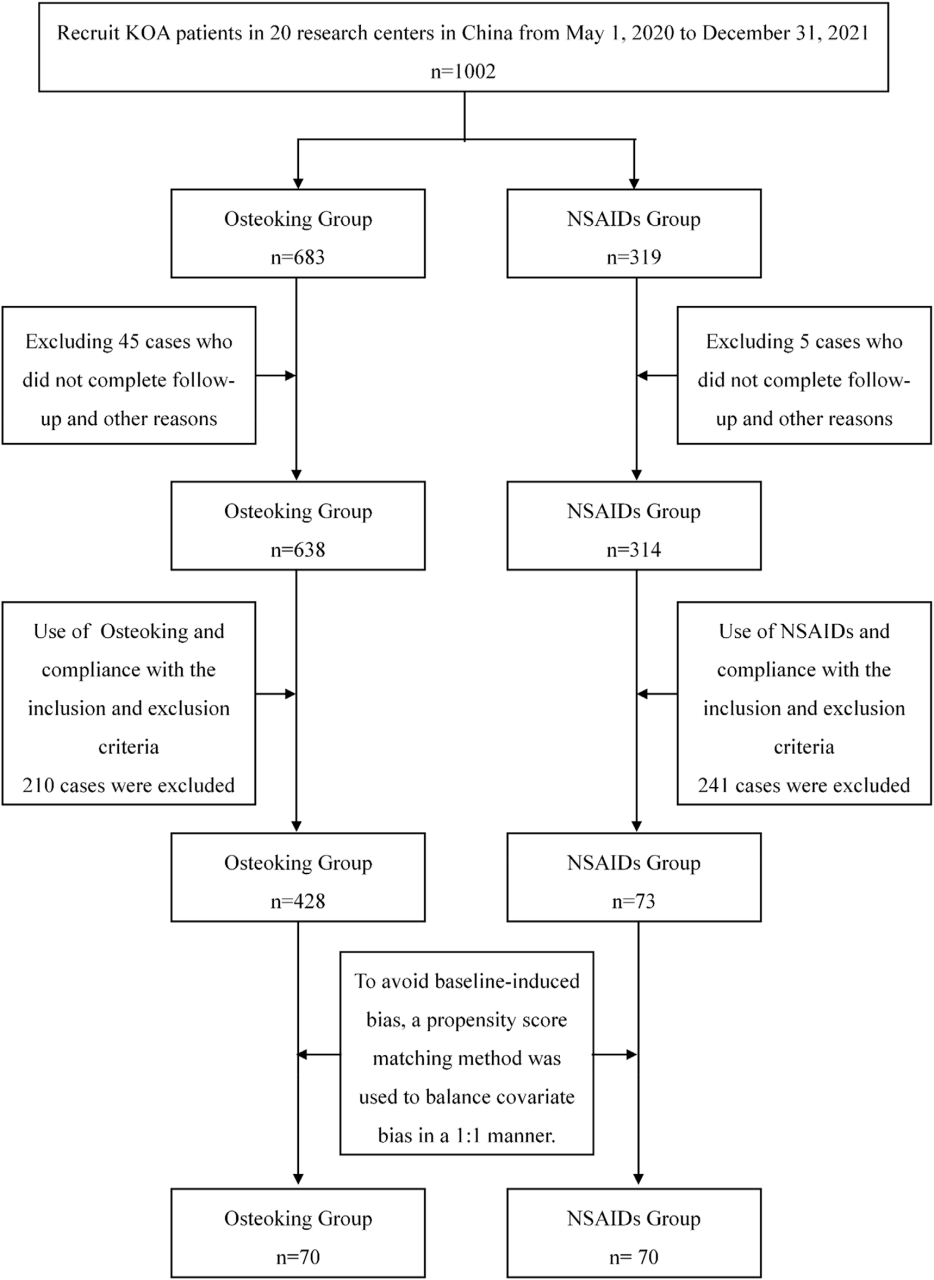
Illustration of the trial process carried out in the current study.

A total of 1002 participants were recruited for this study, and 952 participants completed follow-up. Among them, 501 patients met the inclusion and exclusion criteria, including 428 patients in the Osteoking treatment group and 73 patients in the NSAIDs treatment group. There were significant differences in age, body weight, BMI, VAS before treatment, WOMAC pain, WOMAC stiffness, WOMAC function, WOMAC total score, and health assessment score between the two groups. To avoid baseline-induced bias, the PSM method was used to perform 1:1 matching to balance covariate bias. A total of 70 patients using Osteoking (Osteoking group) and 70 patients using NSAIDs (NSAIDs group) were enrolled. The baseline data are provided in Table 1, the trial process is shown in Figure 1, and the baseline distribution of cases before and after propensity matching is shown in Supplementary Material S6, 7.

**TABLE 1:** The baseline characteristics before and after Propensity Score Matching (PSM).

Variable	Before PSM					After PSM				
	Total (*n* = 501)	Osteoking group (*n* = 428)	NSAIDs group (*n* = 73)	Statistic	P	Total (*n* = 140)	Osteoking group (*n* = 70)	NSAIDs group (*n* = 70)	Statistic	P
Gender				χ^2^ = 0.071	0.790				χ^2^ = 0.033	0.856
Male	158 (31.54)	134 (31.31)	24 (32.88)			45 (32.14)	23 (32.86)	22 (31.43)		
Female	343 (68.46)	294 (68.69)	49 (67.12)			95 (67.86)	47 (67.14)	48 (68.57)		
Age	62.00 (55.00, 71.00)	62.00 (55.00, 71.00)	66.00 (58.00, 74.00)	Z = −2.229	0.026	65.00 (56.00, 73.00)	63.00 (55.00, 73.00)	66.50 (58.00, 74.00)	Z = −1.147	0.251
Height	162.00 (158.00, 168.00)	162.00 (158.00, 169.00)	162.00 (157.00, 167.00)	Z = −1.054	0.292	160.00 (155.00, 168.00)	160.00 (155.00, 169.00)	162.00 (157.00, 166.00)	Z = −0.632	0.527
Weight	62.00 (54.00, 69.00)	62.00 (54.00, 70.00)	59.00 (52.00, 65.00)	Z = −2.749	0.006	58.00 (52.00, 64.00)	55.65 (50.00, 64.00)	58.50 (52.00, 65.00)	Z = −0.822	0.411
BMI	23.17 (21.15, 25.01)	23.30 (21.25, 25.48)	22.77 (20.24, 24.28)	Z = −2.462	0.014	22.17 (20.13, 24.22)	21.55 (20.05, 23.97)	22.77 (20.21, 24.34)	Z = −0.709	0.479
Occupation				χ^2^ = 1.963	0.161				χ^2^ = 0.476	0.490
Non-physical worker	230 (45.91)	202 (47.20)	28 (38.36)			56 (40)	30 (42.86)	26 (37.14)		
Physical worker	271 (54.09)	226 (52.80)	45 (61.64)			84 (60)	40 (57.14)	44 (62.86)		
Smoke				χ^2^ = 0.137	0.711				χ^2^ = 0.000	1.000
No	465 (92.81)	398 (92.99)	67 (91.78)			131 (93.57)	66 (94.29)	65 (92.86)		
Yes	36 (7.19)	30 (7.01)	6 (8.22)			9 (6.43)	4 (5.71)	5 (7.14)		
Drink				χ^2^ = 0.446	0.504				χ^2^ = 0.000	1.000
No	468 (93.41)	398 (92.99)	70 (95.89)			134 (95.71)	67 (95.71)	67 (95.71)		
Yes	33 (6.59)	30 (7.01)	3 (4.11)			6 (4.29)	3 (4.29)	3 (4.29)		
KL gread				χ^2^ = 4.495	0.106				χ^2^ = 2.433	0.296
Ⅰ	97 (19.36)	89 (20.79)	8 (10.96)			20 (14.29)	12 (17.14)	8 (11.43)		
Ⅱ	263 (52.5)	218 (50.93)	45 (61.64)			77 (55)	34 (48.57)	43 (61.43)		
Ⅲ	141 (28.14)	121 (28.27)	20 (27.40)			43 (30.71)	24 (34.29)	19 (27.14)		
VAS score	6.00 (5.00, 6.00)	6.00 (5.00, 6.00)	6.00 (5.00, 7.00)	Z = −4.050	<0.001	6.00 (5.00, 7.00)	6.00 (5.00, 7.00)	6.00 (5.00, 7.00)	Z = −0.341	0.733
WOMAC pain score	20.00 (14.00, 24.00)	18.00 (13.00, 24.00)	23.00 (21.00, 26.00)	Z = −4.535	<0.001	22.00 (20.00, 26.00)	22.00 (18.00, 26.00)	23.00 (21.00, 26.00)	Z = −0.434	0.664
WOMAC stiffness score	5.00 (2.00, 8.00)	5.00 (2.00, 8.00)	2.00 (0.00, 6.00)	Z = −4.238	<0.001	2.00 (0.00, 6.00)	2.00 (0.00, 6.00)	2.00 (0.00, 6.00)	Z = −0.321	0.748
WOMAC physical function score	68.00 (48.00, 80.00)	66.00 (46.00, 80.00)	76.00 (62.00, 83.00)	Z = −2.726	0.006	74.00 (60.00, 83.00)	73.00 (59.00, 81.00)	76.00 (62.00, 83.00)	Z = −0.565	0.572
WOMAC total score	93.00 (66.00, 109.00)	90.50 (63.00, 109.00)	103.00 (90.00, 111.00)	Z = −2.696	0.007	100.00 (82.00, 111.00)	95.00 (81.00, 112.00)	102.00 (89.00, 110.00)	Z = −0.604	0.546
EuroQol5D-3L	0.54 (0.54, 0.63)	0.54 (0.54, 0.65)	0.54 (0.40, 0.54)	Z = −1.363	0.173	0.54 (0.40, 0.54)	0.54 (0.40, 0.54)	0.54 (0.40, 0.54)	Z = −1.467	0.142
EuroQol VAS	50.00 (45.00, 65.00)	55.00 (45.00, 68.00)	45.00 (40.00, 60.00)	Z = −3.129	0.002	45.00 (44.00, 60.00)	50.00 (41.00, 60.00)	45.00 (45.00, 60.00)	Z = −0.080	0.936

Normally distributed data are presented as mean ± sd, Non-normally distributed data are presented as median (Q1, Q3).

### 3.2 The primary outcome measure

**FIGURE 2:**
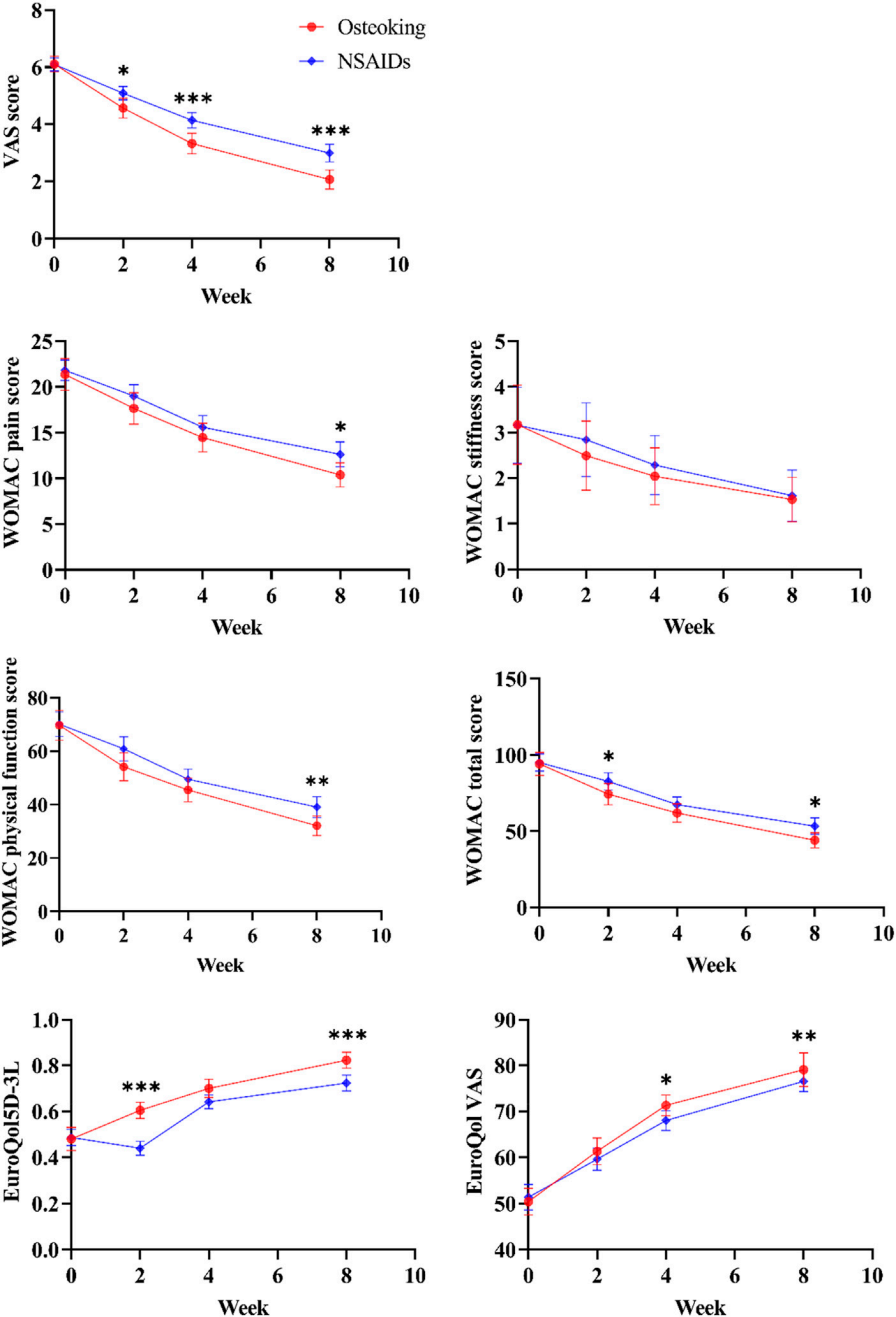
Shows the overall trend of VAS, WOMAC, EuroQol5D-3L and EuroQol VAS scores.

The VAS scores were improved significantly by the treatment of both Osteoking and NSAID (*p* < 0.001). Notably, After 8 weeks of treatment, the VAS score of the Osteoking treatment group after 8 weeks was effectively reduced to 2.00 (1.00, 3.00), which was lower than that of the NSAIDs treatment group’s 3.00 (2.00, 4.00) (*p* < 0.001). Moreover, from the 2 weeks of treatment, the VAS scores of the two groups showed significant differences, and this trend continued until the 8 weeks Table 2 and Figure 2, Supplementary Material S8.

**TABLE 2:** Primary and Secondary outcomes at 8 Weeks.

Variable	Total (*n* = 140)	Osteoking group (*n* = 70)	NSAIDs group (*n* = 70)	Statistic	P
VAS score	2.00 (1.00, 3.00)	2.00 (1.00, 3.00)	3.00 (2.00, 4.00)	Z = −3.971	<0.001
WOMAC pain score	11.00 (8.00, 14.00)	10.00 (8.00, 13.00)	11.00 (8.00, 16.00)	Z = −2.304	0.021
WOMAC stiffness score	0.00 (0.00, 3.00)	1.00 (0.00, 3.00)	0.00 (0.00, 3.00)	Z = −0.821	0.411
WOMAC physical function score	34.00 (26.00, 43.00)	32.00 (23.00, 39.00)	39.07 ± 16.45	Z = −2.622	0.009
WOMAC total score	46.00 (34.00, 60.00)	44.00 (31.00, 55.00)	53.31 ± 22.47	Z = −2.499	0.012
EuroQol5D-3L	0.81 (0.63, 0.91)	0.91 (0.73, 0.91)	0.73 (0.63, 0.83)	Z = −4.390	<0.001
EuroQol VAS	80.00 (70.00, 85.00)	80.00 (79.00, 90.00)	80.00 (70.00, 84.00)	Z = −2.671	0.008

Normally distributed data are presented as mean ± sd, Non-normally distributed data are presented as median (Q1, Q3).

### 3.3 The secondary outcome measure

The intra-group multiple comparisons of WOMAC scores at each time point showed a significant improvement in both Osteoking and NSAIDs treatment groups according to the comparison between before and after the treatment (*p* < 0.001). All the WOMAC pain score, WOMAC physical function score and WOMAC total score were reduced by the treatment of Osteoking for 8 weeks, which was lower than those in the NSAIDs treatment group (*p* < 0.05), but there was no difference in the improvement of WOMAC stiffness score between the Osteoking treatment group and the NSAIDs treatment group (*p* > 0.05). Table 2 and Figure 2, Supplementary Material S9.

The intra-group multiple comparisons of EQ-5D-3L and EQ-VAS at each time point both showed a significant improvement by the treatment of Osteoking and NSAIDs (*p* < 0.001). After 8 weeks of treatment, both the EQ-5D-3L and EQ-VAS in the Osteoking treatment group were higher than those in the NSAIDs treatment group (*p* < 0.05). Moreover, from the 4 weeks of treatment, the EQ-VAS of the two groups showed significant differences, and this trend continued until the 8 weeks Table 2 and Figure 2, Supplementary Material S10.

The values shown are the least squares mean values calculated based on the generalized linear mixed model, and there was a significant improvement in both groups after and before treatment (*p* < 0.001).

Independent sample *t*-test or rank sum test was conducted between the two groups, *p < 0.05, **p < 0.01, ***p < 0.001.

### 3.4 Subgroup analysis

**FIGURE 3:**
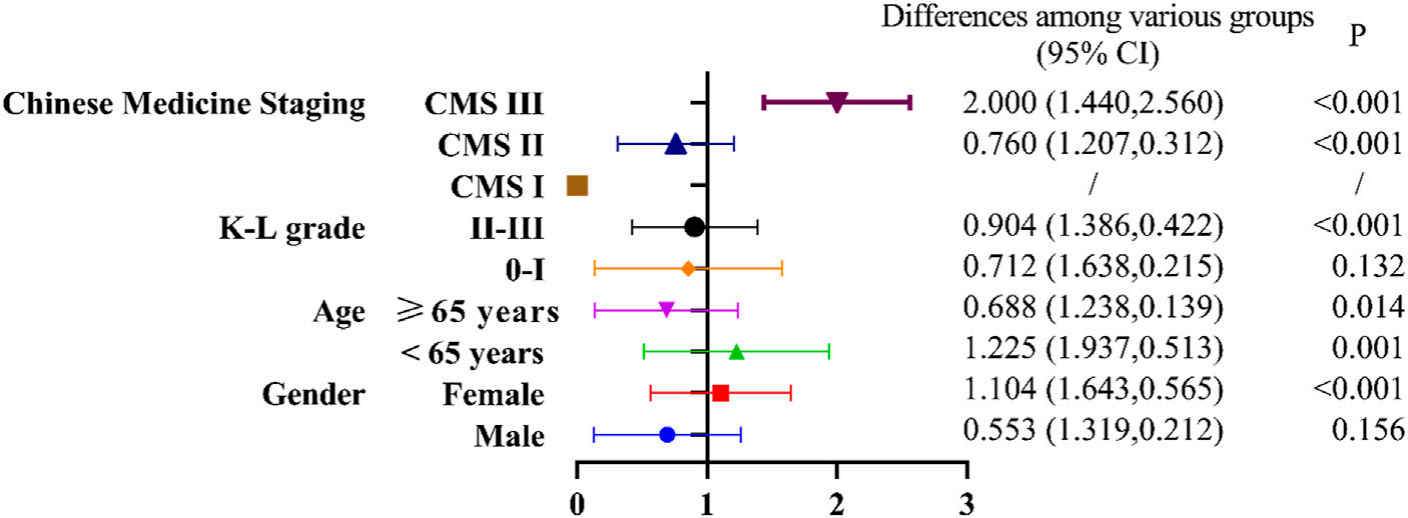
Forest plot of subgroup analysis among the Osteoking treatment group and the NSAIDs treatment group.

For subgroup analysis, we stratified patients by age, gender, K-L grade, and Chinese Medicine staging (CMS), and then compared the differences in VAS scores in different subgroups after 8 weeks of treatment using GLMM. As shown in Figure 3, the KOA patients with CMS III, the NSAIDs treatment group was better than the Osteoking treatment group (*p* < 0.001). Regarding to the KOA patients with CMS II, the improvement on VAS scores in the Osteoking treatment group was better than that in the NSAIDs treatment group (*p* < 0.001). Due to the small sample size of the KOA patients with CMS I, GLMM statistics could not be performed. In addition, the improvement on VAS scores in the Osteoking treatment group was better than that in the NSAIDs treatment group for all KOA patients with K-L Ⅱ-Ⅲ grades (*p* < 0.001), but there was no difference in clinical efficacy of Osteoking between the two groups with K-L 0-Ⅰ grade. Moreover, the clinical efficacy of Osteoking treatment in KOA patient over 65 years old was better (*p* < 0.05), while that of NSAIDs treatment in KOA patients under 65 years old was better (*p* < 0.01). Interestingly, NSAIDs treatment was more suitable for female patients (*p* < 0.001), while the two treatment showed similar clinical efficacy in male patients.

Taking the NSAIDs treatment group as the coefficient 1, under the premise of *p* < 0.05, B < 1 indicates that the efficacy of the Osteoking treatment group is weaker than that of the NSAIDs treatment group, and B > 1 indicates that the efficacy of the Osteoking treatment group is superior to that of the NSAIDs treatment group. Stratification was performed by different subgroups. The differences among various groups were calculated using generalized linear mixed model based on the least squares mean, with a 95% confidence interval in parentheses. The *p*-value was corrected to a significance level of 0.05 using Bonferroni’s method.

### 3.5 Safety outcomes

A total of 14 adverse events occurred in 501 subjects (2.79%), including 5 adverse reactions (1.00%). All subjects did not stop taking the drug and completed follow-up. Among them, 13 adverse events occurred in the Osteoking treatment group (3.04%), including 5 adverse reactions (1.17%); one adverse event occurred in the NSAIDs treatment group (1.37%), with no drug-related adverse reactions. There was no difference in the incidence of adverse events and adverse reactions between the two groups (*p* > 0.05). No serious adverse events occurred in either group. Table 3, Supplementary Material S11.

**TABLE 3:** Comparison of adverse events (adverse reactions) between the two groups.

Variable	Total (*n* = 501)	Osteoking group (*n* = 428)	NSAIDs group (*n* = 73)	X^2^	P
adverse events	14 (2.79)	13 (3.04)	1 (1.37)	0.638	0.424
serious adverse event	0	0	0	—	—
adverse reaction	5 (1.00)	5 (1.17)	0	0.861	0.353

## 4 Discussion

KOA has become a worldwide medical problem due to its high incidence rate, high disability rate, high health hazards and high economic burden. It is of great significance to study the clinical efficacy characteristics and advantages of drugs in the treatment of KOA for guiding clinical rational drug use. This national trial is the first study to compare the effectiveness and safety of using Osteoking and non steroidal anti-inflammatory drugs in the treatment of Chinese patients with KOA. At present, drug therapy for KOA mainly includes NSAIDs, glucosamine, and sodium hyaluronate injection. Among them, NSAIDs are recognized as first-line KOA drugs, but there are gastrointestinal and cardiovascular safety risks (Park et al., 2023). In recent years, more and more evidence shows that the treatment of traditional Chinese medicine is effective in preventing and treating KOA, improving clinical efficacy and reducing adverse reactions (Ye et al., 2023), especially for Xianling Gubao Capsule (Wu et al., 2021), Zhuanggujie Capsule (Lu et al., 2018), Jintiange Capsule (Chen Z. et al., 2023), Xiaotong Patch (Guo et al., 2021), Compound Nanxing Zhitong Paste (Wang et al., 2012), Gutong Patch (Wang et al., 2023) and other traditional Chinese patent medicines and simple preparations, which have been recommended in many Chinese clinical guidelines (Joint Surgery Group of Chinese Medical Association, 2021; Standardization project team of traditional Chinese, 2021; Orthopedics and Traumatology Branch of China, 2020; Chen et al., 2016).

As a legacy of thousands of years of Chinese civilization, TCM has gained much attention for its outstanding efficacy in treating KOA. In addition to its therapeutic effect, it also has the characteristic of fewer adverse reactions. Some studies have shown that some traditional Chinese medicines can improve the progression of KOA and protect joint cartilage cells (Xia et al., 2020). Osteoking is composed of 9 kinds of drugs, which have the effects of promoting blood circulation, enriching qi, nourishing liver and kidney, healing bones and muscles, reducing swelling and pain, and promoting fracture healing. Osteoking is mainly used for bone and joint diseases, femoral head necrosis, lumbar disc herniation, and fractures. Acute and long-term toxicity tests showed no abnormal changes in various physiological indicators in animals, indicating that the preparation is safe (Supplementary Material S12–14).Based on the UPLC analysis, five drug monomers were identified, including Astragaloside, Aucubin, Ginsenoside, Notoginsenoside, and Hesperidin (Xia et al., 2020). Modern research has found that astragaloside can inhibit inflammatory factors such as IL-1β, IL-6, TNF-α, NF-κB, and improve cartilage degradation (Li et al., 2019). Aucubin can inhibit the apoptosis of chondrocytes, protect articular cartilage and slow down osteoarthritis (Wang et al., 2019). Ginsenoside can inhibit inflammation and pyroptosis, and has a clear anti-OA effect (Tian et al., 2023; Luan et al., 2022). Notoginsenoside improves OA by inhibiting the PI3K/Akt/NF-κB pathway by down-regulating the expression of inflammatory factor MRNA (Ju et al., 2020). Hesperidin can prevent chondrocyte damage caused by antioxidant effect *in vitro* (Gao et al., 2018), and also has the effect of anti-inflammatory factors (Fu et al., 2018).Osteoking can slow the progression of osteoarthritis by preventing cartilage degeneration, reducing subchondral sclerosis, and improving gait disorders. The mechanism of action of Osteoking in treating KOA may be closely related to TGF-β, TNF-α, and NF-κB cell signaling pathways (Xia et al., 2020). By promoting the secretion of TGF-β1 and participating in TGF-β signaling pathway transduction, it promotes the secretion of extracellular matrix by joint cartilage cells, inhibits cartilage cell apoptosis, increases the adaptability of cartilage cells to mechanical stress, thereby protecting joint cartilage. At the same time, it can also inhibit the activation of inflammatory signaling pathways TNF-α and NF-κB by reducing the expression of RELA and TNFRSF1A, thereby inhibiting inflammatory response and cell apoptosis (Li et al., 2023).

This study is a national prospective case-control study. In order to avoid the bias caused by the baseline in different regions, the propensity score is used for processing. Compared with the results before matching, the components of the main efficacy indicators are almost the same (Supplementary Material S15). This study showed improvement in primary and secondary efficacy evaluations, including VAS, WOMAC, and EQ-5D in all groups. This study demonstrates that both Osteoking and NSAIDs can effectively alleviate pain, improve daily life, and enhance quality of life in KOA patients. Starting from the second week, Osteoking showed a better pain relief effect than NSAIDs, with a more significant advantage at 4 and 8 weeks. Osteoking was superior to NSAIDs in improving pain and difficulty in daily activities in week 8. In terms of morning stiffness, both treatment methods showed significant improvement compared to before treatment, but there was no difference. For KOA patients with elderly or comorbidities, the use of oral nonsteroidal anti-inflammatory drugs is usually limited, as patients with comorbidities are more prone to side effects, and their incidence will gradually increase (Osani et al., 2020). Therefore, Osteoking is an optional traditional Chinese patent medicines and simple preparations with comparable clinical efficacy of NSAIDs, and it has more advantages in improving pain and daily life after 2 weeks.

KOA affects all aspects of daily life, and related deformities can lead to a stiff, unstable, and painful gait, thereby reducing the distance of independent walking, accompanied by weight gain, sleep problems, and depression (Czypionka et al., 2020). As the population ages and obesity problems become increasingly serious in many countries, the economic burden on the healthcare system may be higher in the coming years (Salmon et al., 2016). Most official national pharmacoeconomic evaluation guidelines mention EQ-5D as the preferred tool for determining health utility or as a description of suitable tools (Fawaz et al., 2023). EQ-5D is a preference-based measure of health that is widely used in economic evaluation of health technologies (Brooks, 1996; Hainsworth et al., 2024). The results of this study show that Osteoking has significant advantages over NSAIDs in improving quality of life and perceived health status at 2 and 8 weeks. The use of Osteoking can further improve medical burden.

The symptoms of KOA patients are the main reason for seeking medical treatment. Therefore, our team proposed a staging method based on clinical symptoms, Chinese Medicine staging (CMS). Through a national cross-sectional study, comparing the correlation between CMS and Kellgren-Lawrence grading for treatment, it was found that CMS was more suitable for assessing the severity of symptoms in KOA patients to determine non-surgical treatment, but not as suitable as Kellgren-Lawrence grading for assessing surgical treatment (Chen et al., 2024).In order to find out which type of KOA population Osteoking has a therapeutic advantage, we conducted a subgroup analysis of common influencing factors (gender, age, KL grade) and different clinical stages (Chen et al., 2024; Cao et al., 2023). It is helpful for doctors to make personalized plans for different groups of people in clinical practice. We used stratified research to explore the efficacy characteristics of different populations. We found that for different CMS, NSAIDs are recommended for treatment in CMS III (VAS 8–10), which can effectively alleviate pain. Osteoking is recommended for use in CMS II(VAS 4–7), and its efficacy in improving pain is significantly better than NSAIDs, which may be related to multiple pathways involved in regulating inflammatory response and bone metabolism. For K-L II-III grades, and patients over 65 years old the efficacy of Osteoking is better than NSAIDs. Clinical medication can be based on population characteristics for reference. For patients under 65 years old and female, the efficacy of NSAIDs is better than Osteoking. A total of 14 adverse events occurred in this study, mainly including mild adverse events such as chest tightness, toothache, cold, sore throat, etc., including 5 cases of adverse reactions, which were drowsiness, dizziness, dry throat, dry throat, and dizziness. The clinical judgment was mild adverse reaction. No drug was stopped. There was no significant difference in the incidence of adverse events and non-drug reactions between the two groups, indicating that Osteoking has clear safety in clinical practice.

Although this study controlled confounding factors through prospective design and statistical processing, it still had the common limitations of real-world research. Randomization and blinding are difficult to achieve in real-world research. Compared with randomized controlled trials, there are more confounding factors and biases. Nevertheless, our results do provide preliminary confirmation of the efficacy and advantages of using Osteoking alone for the treatment of KOA. In the future, we will conduct targeted randomized controlled trials and mechanism studies.

## 5 Conclusion

This study shows that using Osteoking alone or NSAIDs alone has therapeutic effects in reducing joint pain and improving the quality of life of KOA patients, with good safety. However, Osteoking has better clinical efficacy in 2–8 weeks. For different characteristics of the population, we recommend Osteoking for patients with CMS II(VAS 4-7), K-L II-III grades, and patients over 65 years old, and NSAIDs for patients with CMS III(VAS 8-10) and patients under 65 years old.

## Data Availability

The raw data supporting the conclusion of this article will be made available by the authors, without undue reservation.
