# Supplementary material for: Clinical efficacy of Osteoking in knee osteoarthritis therapy: a prospective, multicenter, non-randomized controlled study in China
Source: Front Pharmacol. 2024 Jun 28;15:1381936. doi: 10.3389/fphar.2024.1381936 (PMC11239513; doi:10.3389/fphar.2024.1381936)
Supplement: Supplementary file 2 [file Table1.DOCX]

**Table of contents**

[**Supplementary Material 1 Optimal Preparation Method for Osteoking 2**](#_Toc167318261)

[**Supplementary Material 2 Type of Osteoking 4**](#_Toc167318262)

[**Supplementary Material 3 The method of extracting components 5**](#_Toc167318263)

[**Supplementary material 4 KOA subject source distribution and research center information 11**](#_Toc167318264)

[**Supplementary material 5 The Integral System of Utility Values for Quality of Life of Chinese Residents 13**](#_Toc167318265)

[**Supplementary material 6 SMD distribution diagram 14**](#_Toc167318266)

[**Supplementary material 7 Distribution of cases after propensity score matching 15**](#_Toc167318267)

[**Supplementary Material 8 Comparison of VAS scores between two groups at different time points 16**](#_Toc167318268)

[**Supplementary Material 9 Comparison of WOMAC scores between two groups at different time points 17**](#_Toc167318269)

[**Supplementary Material 10 Comparison of EuroQol5D-3L and EuroQol VAS between two groups at different time points 18**](#_Toc167318270)

[**Supplementary Material 11 Incidence of adverse events (including adverse reactions) in the two groups 19**](#_Toc167318271)

[**Supplementary Material 12 Toxicological Study of Osteoking 22**](#_Toc167318272)

[**Supplementary Material 13 Pharmacological Study of Osteoking 32**](#_Toc167318273)

[**Supplementary Material 14 Stability Study of Osteoking 34**](#_Toc167318274)

[**Supplementary Material 15 Comparison of VAS scores between two groups at different time points(before Propensity Score Matching) 48**](#_Toc167318275)

**Supplementary Material 1 Optimal Preparation Method for Osteoking**


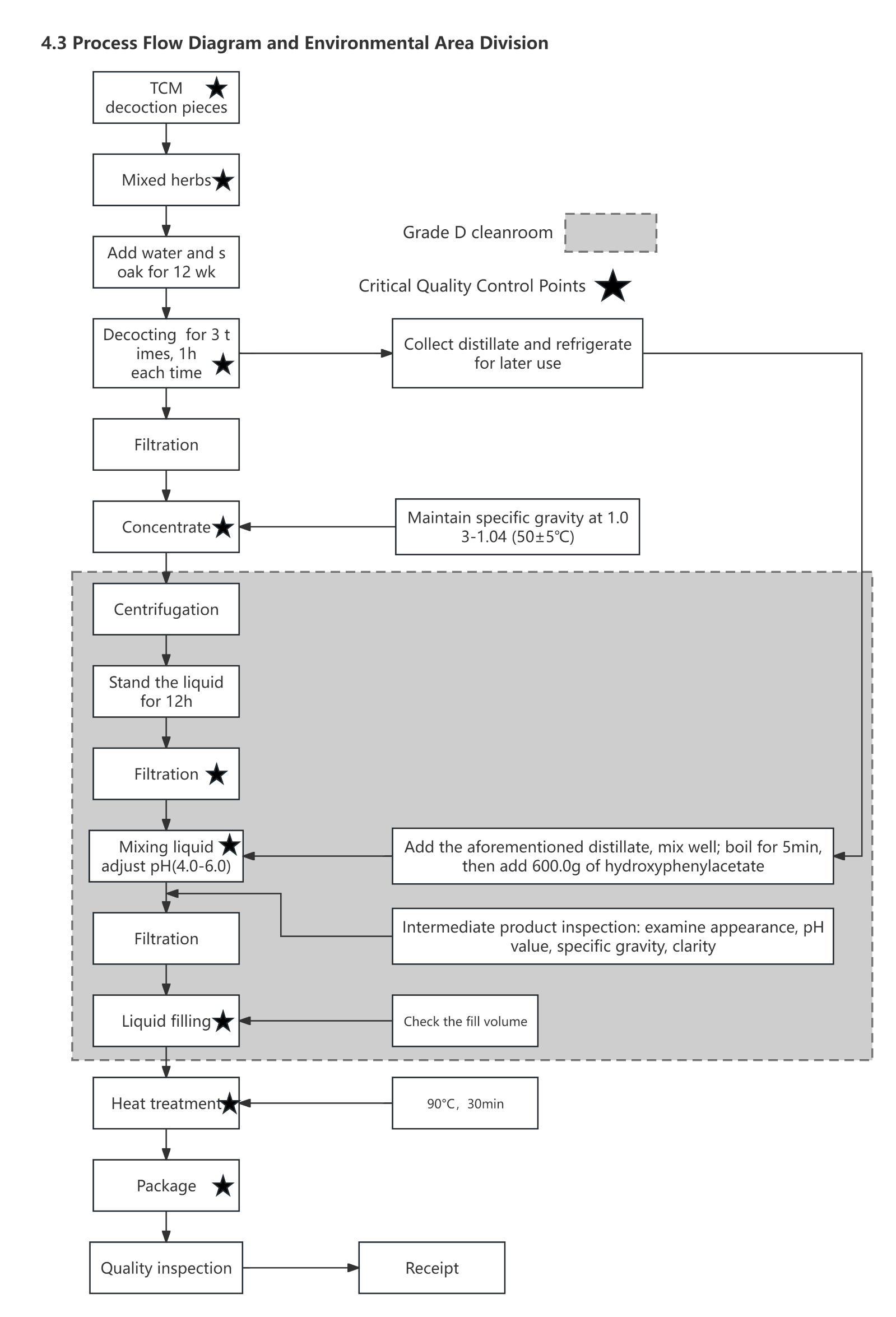


The preparation method is from the Chinese Pharmacopoeia 2020 Edition.

Henggu Gushangyu Heji

【Prescription】Chen Pi 10g Hong Hua 15g

San Qi 30g Du Zhong 30g

Ren Shen 20g Huang Qi 40g

Yang Jin Hua 6g Zuan Di Feng 10g

Bie Jia 10g

【Preparation Method】Take the above nine traditional Chinese medicinal herbs and soak them in water for 12 hours. Boil the soaked herbs three times, each time for 1 hour, and simultaneously collect the distillate and store it in the refrigerator for later use. Combine the decoctions, filter them, and concentrate the filtrate to a relative density of 1.03 ~ 1.04 (at 50℃). Centrifuge the concentrated filtrate and let it stand for 12 hours, then filter it again. Mix the filtered liquid with the previously collected distillate. Add 0.4g of ethyl paraben and adjust the pH to 4.0 ~ 6.0 using a 0.05% sodium bicarbonate solution. Add water to bring the total volume to 1000ml, filter again, and package the final product.

【Description】This product is a brownish-black liquid with a pungent and slightly bitter taste.

**Supplementary Material 2 Type of Osteoking**

From Chinese Pharmacopoeia 2020 Edition

【Description】This product is a brownish-black liquid with a pungent and slightly bitter taste.

【Functions and Indications】Promotes blood circulation and Qi, tonifies the liver and kidneys, connects bones and tendons, reduces swelling, relieves pain, and promotes fracture healing. Used for fresh and old fractures, femoral head necrosis, osteoarthritis, and lumbar disc herniation.

【Administration and Dosage】

- Route: Oral administration.

- Dosage:

- Adults: 25ml per dose.

- Children aged 6 to 12 years: 12.5ml per dose.

- Frequency: Take once every two days.

- Timing: Take one hour after meals.

- Course of Treatment: 12 days constitute one treatment course.

【Precautions】

(1) Patients with fractures should use the medication only after reduction and fixation; (2)Used with caution in individuals with heart, lung, or kidney insufficiency; (3)Contraindicated for those with a history of mental illness, glaucoma, or pregnancy; (4)Some patients may experience dry mouth and mild dizziness after taking the medication, which can resolve on its own.

【Specifications】Each bottle contains (1) 12.5ml, (2) 25ml, or (3) 50ml.

【Storage】Store in a sealed container in a cool place.

**Supplementary Material 3 The method of extracting components**

**(1) Active principle**

TABLE 1 | The composition of Osteoking

| Chinese herb | Full taxonomy name | Latin name | Wight(g) | Parts used |
| --- | --- | --- | --- | --- |
| Chen pi | Citrus reticulata blanco | Pericarpium citri reticulatae | 10 | Peel |
| Hong hua | Carthamus tinctorius L | Flos carthami | 15 | Flower |
| San qi | Panax notoginseng (burkill) F.H.Chen | Radix notoginseng | 30 | Root |
| Du zhong | Eucommia ulmoides oliv | Cortex eucommiae | 30 | Bark |
| Ren shen | Panax ginseng C.A.Mey | Radix ginseng | 20 | Root |
| Huang qi | Astragalus mongholicus bunge | Radix astragali | 40 | Root |
| Yang jin hua | Datura metel L | Flos daturae | 6 | Flower |
| Zuan di feng | Schizophragma integrifolium (Franch.)Oliv | Schizophragma integrifolium | 25 | Root; Stem |
| Bie jia | Carapax trionycis | Trionyx sinensis carapace | 10 | Carapace |

**(2) Source of drug information**

Chen pi, Citrus reticulata Blanco [Rutaceae]: 30g, https://mpns.science.kew.org/mpns-portal/plantDetail?plantId=2724336&query=Pericarpium+citri+reticulatae&filter=&fuzzy=false&nameType=all&dbs=wcsCmp

Hong hua, Carthamus tinctorius L. [Asteraceae]: 30g, https://mpns.science.kew.org/mpns-portal/plantDetail?plantId=2900984&query=carthamus+tinctorius&filter=&fuzzy=false&nameType=all&dbs=wcsCmp

San qi, Panax notoginseng (Burkill) F.H.Chen [Araliaceae]: 30g, https://mpns.science.kew.org/mpns-portal/plantDetail?plantId=146751&query=panax+notoginseng&filter=&fuzzy=false&nameType=all&dbs=wcs

Du zhong, Eucommia ulmoides Oliv. [Eucommiaceae]: 30g, https://mpns.science.kew.org/mpns-portal/plantDetail?plantId=514395&query=eucommia+ulmoides&filter=&fuzzy=false&nameType=all&dbs=wcs

Ren shen, Panax ginseng C.A.Mey. [Araliaceae]: 20g, https://mpns.science.kew.org/mpns-portal/plantDetail?plantId=146697&query=panax+ginseng&filter=&fuzzy=false&nameType=all&dbs=wcs

Huang qi, Astragalus mongholicus Bunge [Fabaceae]: 40g, https://mpns.science.kew.org/mpns-portal/plantDetail?plantId=2661222&query=astragalus+mongholicus&filter=&fuzzy=false&nameType=all&dbs=wcs

Yang jin hua, Datura metel L. [Solanaceae]: 15g, <https://mpns.science.kew.org/mpns-portal/plantDetail?plantId=2757816&query=datura+metel&filter=&fuzzy=false&nameType=all&dbs=wcsCmp>

**Datura metel L. [Solanaceae], known in Chinese as "yang jin hua" is a botanical medicine. This substance is included in the Pharmacopoeia of the People's Republic of China: 2020 edition, with a recommended dosage of 0.3-0.6g per day. Here are the detailed information， https://ydz.chp.org.cn/#/item?bookId=1&entryId=421. The Osteoking used in this study contains 6g of prepared yang jin hua per 1000ml, with an oral administration of 25ml every 2 days, for a 12-day treatment period, totaling two courses. A total of 300ml of Osteoking is consumed, with the daily intake significantly lower than the dosage specified in the pharmacopoeia, ensuring it is within a safe range.**

Zuan di feng, Schizophragma integrifolium Oliv, a synonym of Hydrangea ampla (Chun) Y.De Smet & Granados [Hydrangeaceae]: 25g, https://mpns.science.kew.org/mpns-portal/plantDetail?plantId=3009446&query=Schizophragma+integrifolium+Oliv.&filter=&fuzzy=false&nameType=all&dbs=wcsCmp, https://powo.science.kew.org/taxon/urn:lsid:ipni.org:names:794782-1.

Bie jia, Carapax Trionycis, The back shell of the animal Trionyx sinensis Wiegmann of family Trionychidae: 10g. The drug Carapax Trionycis mentioned in this study is the shell of Trionyx sinensis Wiegmann. Trionyx sinensis Wiegmann is an animal that has been standardized and artificially bred for a long time in China. The following is detailed information about Trionyx sinensis Wiegmann: https://marinespecies.org/traits/aphia.php?p=taxdetails&id=1377599#links

**(3) Preparation and UPLC Analysis of Osteoking**

All Osteoking used in this study were purchased from the Yunnan Crystal Natural Pharmaceutical Co., Ltd. (Kunming, China) (lot. No. 20190330). The relative proportions are shown in Table 1. The identification of all the plant materials used in this study was undertaken by Yunnan Crystal Natural Pharmaceutical Co., Ltd. according to the Chinese Pharmacopeia (2015, Edition). Ultra-Performance Liquid Chromatograph (UPLC) was utilized to control the quality of Osteoking and identify the accurate chemical component. Five chromatogram peaks that represent five drug monomers respectively were identified in UPLC and showed in Figure[1].

[1]Ling H, Zeng Q, Ge Q, Chen J, Yuan W, Xu R, Shi Z, Xia H, Hu S, Jin H, Wang P, Tong P. Osteoking Decelerates Cartilage Degeneration in DMM-Induced Osteoarthritic Mice Model Through TGF-β/smad-dependent Manner. Front Pharmacol. 2021 Jun 15;12:678810. doi: 10.3389/fphar.2021.678810. PMID: 34211396; PMCID: PMC8239307.


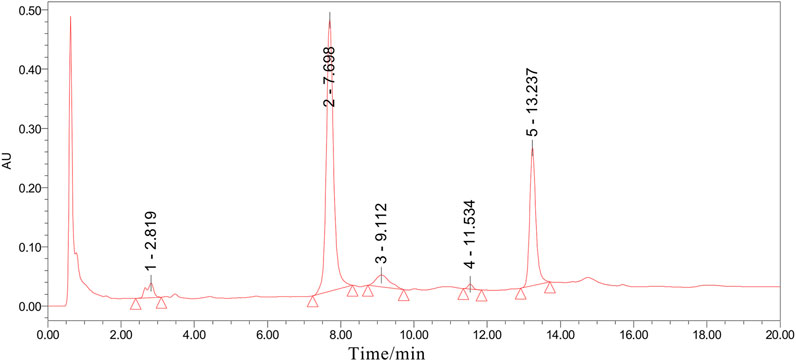


The UPLC analysis result of Osteoking solution. All chromatographic peak signaling was recorded at 260 nm and the peak area was integrated under the instrument’s protocol. Peak 1–5 respectively indicated Astragaloside, Aucubin, Ginsenoside, Notoginsenoside, and Hesperidin.

**(4) Identification**

From Chinese Pharmacopoeia 2020 Edition

①Take 50ml of the product and extract it with ether twice, each time using 40ml of ether. Discard the ether layer. Extract the water layer with ethyl acetate twice, each time using 20ml of ethyl acetate. Reserve the water layer. Combine the ethyl acetate extracts and evaporate the solvent to dryness. Dissolve the residue in 0.5ml of methanol to prepare the test solution. Take 1g of Chen Pi reference material and extract it with 40ml of ether using ultrasonic treatment for 10 minutes. Discard the ether layer. Evaporate the ether from the residue, then extract it with 20ml of ethyl acetate using ultrasonic treatment for 20 minutes. Filter and evaporate the solvent to dryness. Dissolve the residue in 1ml of methanol to prepare the reference material solution. Prepare a saturated solution of hesperidin reference substance in methanol as the reference substance solution. According to the Thin Layer Chromatography method (General Rule 0502), take 10μl of each of the above three solutions and spot them on the same TLC plate coated with silica gel G containing 0.5% sodium carboxymethylcellulose as a binder. Use ethyl acetate-methanol-water (100:17:13) as the developing solvent for the first development. Develop the plate over a distance of 3cm, remove it, and dry it. Use the upper layer of toluene-ethyl acetate-formic acid-water (20:101:1) as the developing solvent for the second development. Develop the plate over a distance of approximately 8cm, remove it, and dry it. Spray the plate with aluminum chloride solution and dry it. Examine under UV light (365nm). In the chromatogram of the test solution, fluorescent spots of the same color should appear at the same positions as those in the chromatograms of the reference material and the reference substance.

②Take the reserved aqueous solution from the ethyl acetate extraction in the Identification (1) step. Make the solution alkaline by adding ammonia solution. Extract the alkaline solution with chloroform twice, each time using 20ml of chloroform. Reserve the alkaline aqueous solution. Combine the chloroform extracts and evaporate the solvent to dryness. Dissolve the residue in 0.5ml of methanol to prepare the test solution. Prepare a mixed solution of atropine sulfate reference substance and scopolamine hydrobromide reference substance in methanol, each containing 1mg per ml, to prepare the reference substance solution. According to the Thin Layer Chromatography method (General Rule 0502), take 10μl of each of the above two solutions and spot them on the same TLC plate coated with silica gel G. Use ethyl acetate-methanol-concentrated ammonia solution (8.5:1:0.5) as the developing solvent, pre-saturating the chamber for 20 minutes before developing. Develop the plate, remove it, and dry it. Spray the plate with dilute potassium iodobismuthate solution and examine it under daylight. In the chromatogram of the test solution, spots of the same color should appear at the same positions as those in the chromatogram of the reference substance.

③Take the reserved alkaline aqueous solution from Identification (2). Extract it with water-saturated n-butanol twice, each time using 20ml of n-butanol. Combine the n-butanol extracts. Wash the combined n-butanol extracts with 15ml of water and discard the water wash. Evaporate the n-butanol solvent to dryness. Dissolve the residue in 1ml of methanol to prepare the test solution. Prepare a solution of astragaloside IV reference substance in methanol, with a concentration of 1mg per ml, to use as the reference solution. According to the Thin Layer Chromatography method (General Rule 0502), take 5μl of each of the above two solutions and spot them on the same TLC plate coated with silica gel G. Use chloroform-methanol-water (13:7:2) lower phase solution, which has been left below 10℃ overnight, as the developing solvent. Develop the plate, remove it, and dry it. Spray the plate with 10% sulfuric acid ethanol solution and heat until the spots are clearly visible. Examine the plate under daylight and UV light (365nm). In the chromatogram of the test solution, spots of the same color should appear at the same positions as those in the chromatogram of the reference substance, showing the same color under daylight and the same fluorescent spots under UV light.

④Prepare a mixed solution containing ginsenoside Rb1, ginsenoside Rg1, and notoginsenoside R1 reference substances in methanol, each with a concentration of 1mg per ml, to use as the reference solution. According to the Thin Layer Chromatography method (General Rule 0502), take 5μl each of the test solution from Identification (3) and the reference solution. Spot both solutions on the same high-efficiency silica gel G TLC plate. Use chloroform-ethyl acetate-methanol-water (15:40:22:10) lower phase solution, which has been left below 10℃, as the developing solvent. Develop the plate, remove it, and dry it. Spray the plate with 10% sulfuric acid ethanol solution and heat until the spots are clearly visible. Examine the plate under daylight and UV light (365nm). In the chromatogram of the test solution, spots of the same color should appear at the same positions as those in the chromatogram of the reference substances, showing the same color under daylight and the same fluorescent spots under UV light.

⑤Take 50ml of the product and evaporate it to dryness on a water bath. Dissolve the residue in 15ml of water and pass it through a D101 macroporous adsorption resin column (column diameter 2cm, column height 15cm). Elute the column first with 60ml of water and discard the eluent. Then, elute with 100ml of 10% ethanol and collect the eluent. Evaporate the solvent from the collected eluent to dryness. Dissolve the residue in 3ml of anhydrous ethanol and centrifuge it. Use the supernatant as the test solution. Take 0.5g of Hong Hua reference material, add 30ml of water, and treat it with ultrasound for 30 minutes. Centrifuge and take the supernatant. Process it in the same way as the test solution starting from the "pass through the D101 macroporous adsorption resin column" step to prepare the reference material solution. According to the Thin Layer Chromatography method (General Rule 0502), take 10μl each of the test solution and the reference material solution. Spot both solutions on the same silica gel H TLC plate. Use the upper layer of n-butanol-glacial acetic acid-water (4:1:5) as the developing solvent. Develop the plate, remove it, and dry it. Examine the plate under UV light (365nm). In the chromatogram of the test solution, fluorescent spots of the same yellow color should appear at the same positions as those in the chromatogram of the reference material under UV light.

**(5) Content Determination**

From Chinese Pharmacopoeia 2020 Edition

The content is determined using high-performance liquid chromatography (HPLC) according to General Rule 0512.

Chromatographic Conditions and System Suitability Test:

- Column: Octadecylsilane bonded silica gel (C18 column)

- Mobile Phase: Acetonitrile - 0.1% phosphoric acid solution (21:79)

- Detection Wavelength: 283 nm

- Theoretical Plate Number: The number of theoretical plates calculated for the hesperidin peak should not be less than 5000.

Preparation of Reference Solution: Accurately weigh an appropriate amount of hesperidin reference substance and dissolve it in methanol to prepare a solution containing 25μg of hesperidin per ml.

Preparation of Test Solution: Accurately measure 5ml of the product and place it in a 25ml volumetric flask. Add the mobile phase to the mark, mix well, filter, and use the filtrate as the test solution.

Determination: Accurately inject 10μl each of the reference solution and the test solution into the HPLC system for analysis.

The product should contain no less than 0.10mg of hesperidin (C28H34O15) per ml calculated based on Chen Pi.

# Supplementary material 4 KOA subject source distribution and research center information

| HosPital Number | HosPital Grade | Hospital name | Hospital type | Province | Area | Osteoking Group | NSAIDs Group |
| --- | --- | --- | --- | --- | --- | --- | --- |
| NO.1 | 3A | Peking Union Medical College Hospital, Chinese Academy of Medical Sciences | Western medicine | Beijing | North China | 11 | 0 |
| NO.2 | 3A | Wangjing Hospital of the Chinese Academy of Traditional Chinese Medicine | Traditional Chinese Medicine | Beijing | North China | 12 | 0 |
| NO.3 | 2A | Hunan Aerospace Hospital | Western medicine | Hunan Guipei | Central China | 23 | 2 |
| NO.5 | 3A | Yueyang Second People's Hospital | Western medicine | Yueyang, Hunan | Central China | 6 | 10 |
| NO.6 | 2A | Weihai Weihai People's Hospital | Western medicine | Weihai, Shandong | East China | 11 | 2 |
| NO.7 | 3A | Weihai Municipal Hospital | Western medicine | Weihai, Shandong | East China | 4 | 21 |
| NO.8 | 3A | Weifang People's Hospital | Western medicine | Weifang, Shandong | East China | 19 | 0 |
| NO.9 | 3A | Kunming Hospital of Traditional Chinese Medicine | Traditional Chinese Medicine | Kunming, Yunnan | Southwest | 12 | 33 |
| NO.10 | 3A | Yunnan Provincial Hospital of Traditional Chinese Medicine | Traditional Chinese Medicine | Kunming, Yunnan | Southwest | 326 | 153 |
| NO.11 | 3A | The First Affiliated Hospital of Henan University | Western medicine | Kaifeng, Henan | Central China | 10 | 9 |
| NO.12 | 3 | Yongzhou No.3 People's Hospital | Western medicine | Yongzhou, Hunan | Central China | 11 | 6 |
| NO.14 | 3A | Xi'an Hospital of Traditional Chinese Medicine | Traditional Chinese Medicine | Xi'an, Shaanxi | Northwest | 51 | 14 |
| NO.15 | 2A | Yuanjiang People's Hospital | Western medicine | Yuanjiang, Hunan | Central China | 1 | 4 |
| NO.16 | 3A | The Second Affiliated Hospital of Guizhou Medical University | Western medicine | Qiandongnan Miao and Dong Autonomous Prefecture, Guizhou Province | Southwest | 18 | 15 |
| NO.17 | 3A | The Second Affiliated Hospital of Liaoning University of Chinese Medicine | Traditional Chinese Medicine | Shenyang, Liaoning | northeast | 29 | 10 |
| NO.18 | 3A | Shenyang No.5 People's Hospital | Western medicine | Shenyang, Liaoning | northeast | 30 | 10 |
| NO.19 | 3A | Yunnan Provincial Third People's Hospital | Western medicine | Kunming, Yunnan | Southwest | 48 | 23 |
| NO.20 | 3A | Shaanxi University of Chinese Medicine Affiliated Hospital | Traditional Chinese Medicine | Xianyang, Shaanxi | Northwest | 10 | 0 |
| NO.21 | 2A | Xi'an Jiaotong University Chang'an District Hospital | Western medicine | Xi'an, Shaanxi | Northwest | 4 | 2 |
| NO.22 | 3A | Changde No.1 Hospital of Traditional Chinese Medicine | Traditional Chinese Medicine | Changde, Hunan | Central China | 2 | 0 |
| Total |  |  |  |  |  | 638 | 314 |

# Supplementary material 5 The Integral System of Utility Values for Quality of Life of Chinese Residents

| Variable | Definition | Score |
| --- | --- | --- |
| C | At least one dimension is at level 2 or 3 | 0.039 |
| action ability |  |  |
| MO2 | The action ability dimension is at level 2 | 0.099 |
| MO3 | The action ability dimension is at level 3 | 0.246 |
| Self-care |  |  |
| SC2 | Self-care dimension is at level 2 | 0.105 |
| SC3 | Self-care dimension is at level 3 | 0.208 |
| daily activity |  |  |
| UA2 | The daily activity dimension is at level 2 | 0.074 |
| UA3 | The daily activity dimension is at level 3 | 0.193 |
| Pain/discomfort |  |  |
| PD2 | Pain/discomfort dimension is at level 2 | 0.092 |
| PD3 | Pain/discomfort dimension is at level 3 | 0.236 |
| Anxiety/depression |  |  |
| AD2 | Anxiety/depression dimension is at level 2 | 0.086 |
| AD3 | Anxiety/depression dimension is at level 3 | 0.236 |
| N3 | At least one dimension is at level 3 | 0.022 |

The EQ-5D-3L describes the health status of the subject from five dimensions: mobility, self-care, activities of daily living, pain/discomfort, and anxiety/depression. Each dimension can be divided into three levels (no problem, problem, and extreme problem). Using the Chinese residents' quality of life utility value scoring system, the calculation formula is: U=1-(0.039+0.099*MO2+0.105*SC2+0.074*UA2+0.092*PD2+0.086*AD2+0.246*MO3+0.208*SC3+0.193*UA3+0.236*PD3+0.205*AD3+0.022*N3), and the range of health effect values is [-0.149-1]. The upper limit of utility value 1 indicates a completely healthy state, while the lower limit is -0.149, representing the worst health state.

EQ-VAS uses a 20cm vertically placed visual scale, with 100 points at the top as the "best imaginable health state" and 0 points at the bottom as the "worst imaginable health state". Respondents are asked to mark the position on the scale that best fits their current health status.

# Supplementary material 6 SMD distribution diagram


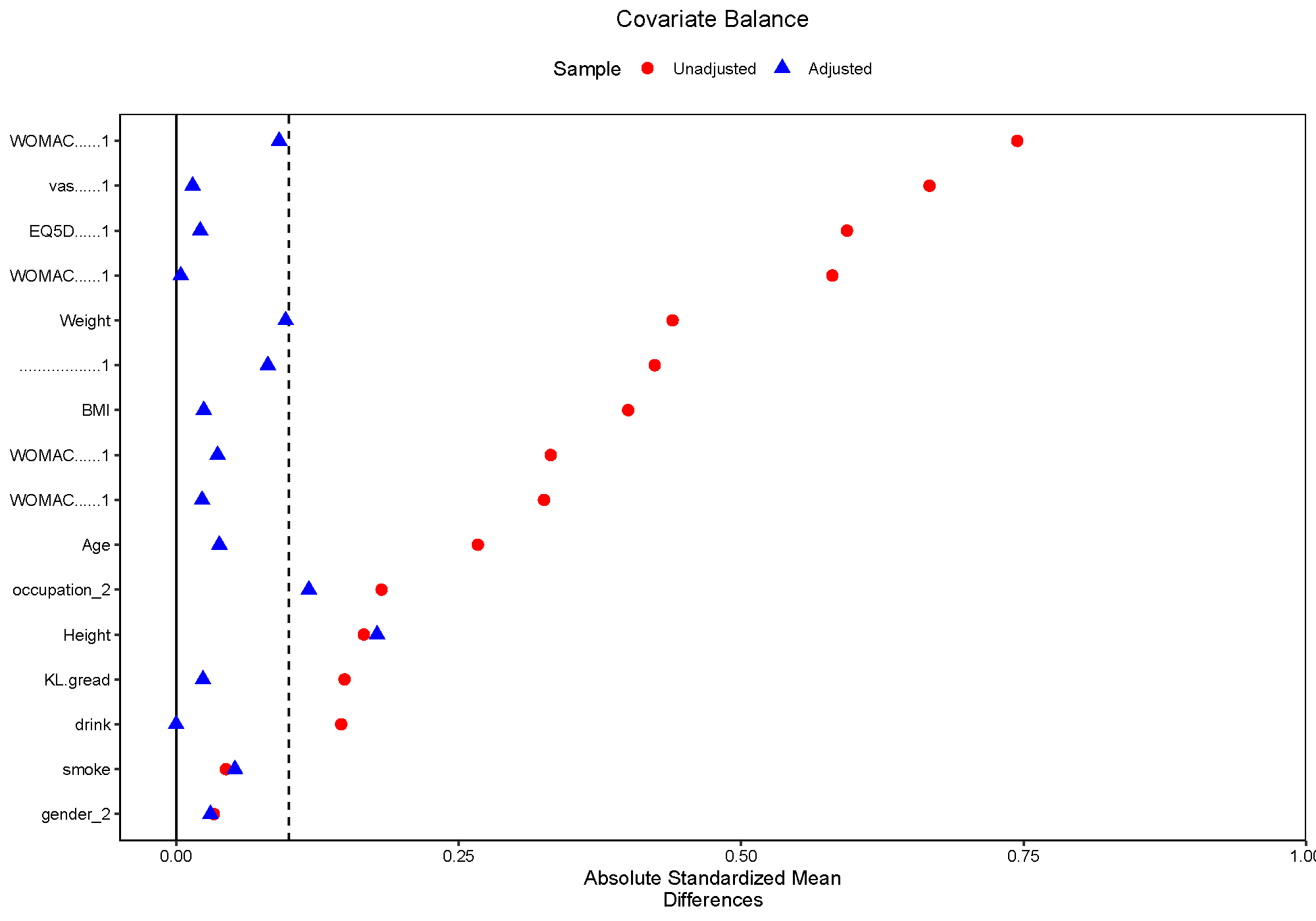


# Supplementary material 7 Distribution of cases after propensity score matching


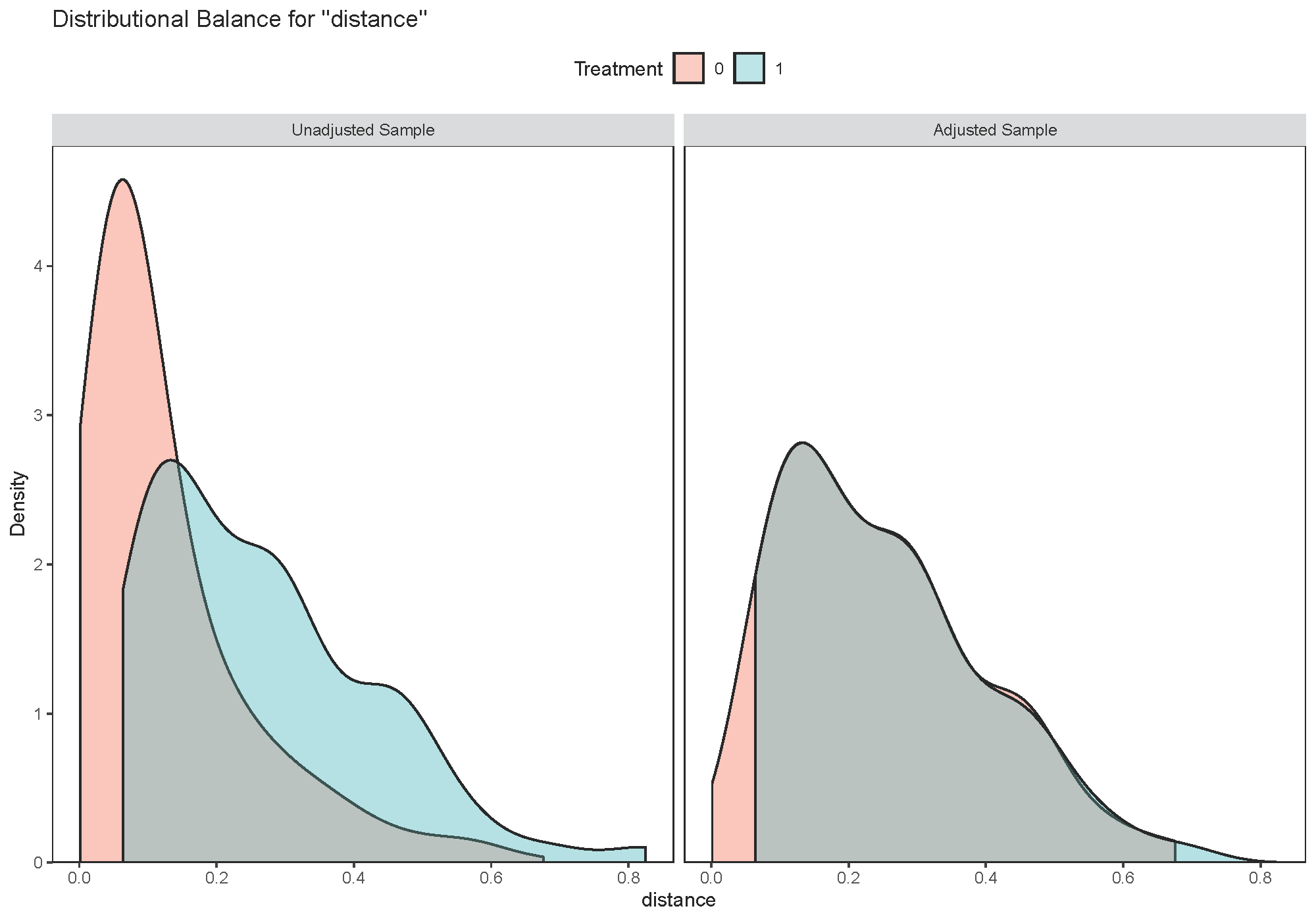


# Supplementary Material 8 Comparison of VAS scores between two groups at different time points

| Variable | Total  (n = 140) | Osteoking Group  (n = 70) | NSAIDs Group  (n = 70) | Statistic | P |
| --- | --- | --- | --- | --- | --- |
| baseline | 6.00 (5.00, 7.00) | 6.00 (5.00, 7.00) | 6.00 (5.00, 7.00) | Z=-0.341 | 0.733 |
| 2 weeks | 5.00 (4.00, 6.00) | 5.00 (4.00, 6.00) | 5.00 (4.00, 6.00) | Z=-2.284 | 0.022 |
| 4 weeks | 4.00 (3.00, 5.00) | 3.00 (2.00, 4.00) | 4.00 (4.00, 5.00) | Z=-3.327 | <0.001 |
| 8 weeks | 2.00 (1.00, 3.00) | 2.00 (1.00, 3.00) | 3.00 (2.00, 4.00) | Z=-3.971 | <0.001 |

# Supplementary Material 9 Comparison of WOMAC scores between two groups at different time points

| Variable | Time | Total  (n = 140) | Osteoking Group  (n = 70) | NSAIDs Group  (n = 70) | Statistic | P |
| --- | --- | --- | --- | --- | --- | --- |
| WOMAC pain score | baseline | 22.00 (20.00, 26.00) | 21.63 ± 6.55 | 23.00 (21.00, 26.00) | Z=-0.434 | 0.664 |
|  | 2 weeks | 18.00 (16.00, 22.00) | 17.56 ± 6.71 | 19.00 (17.00, 23.00) | Z=-1.450 | 0.147 |
|  | 4 weeks | 14.95 ± 5.50 | 14.32 ± 5.64 | 15.59 ± 5.32 | t=-1.362 | 0.176 |
|  | 8 weeks | 11.00 (8.00, 14.00) | 10.00 (8.00, 13.00) | 11.00 (8.00, 16.00) | Z=-2.304 | 0.021 |
| WOMAC stiffness score | baseline | 2.00 (0.00, 6.00) | 2.00 (0.00, 6.00) | 2.00 (0.00, 6.00) | Z=-0.321 | 0.748 |
|  | 2 weeks | 1.00 (0.00, 6.00) | 2.00 (0.00, 5.00) | 1.00 (0.00, 6.00) | Z=-0.095 | 0.924 |
|  | 4 weeks | 1.00 (0.00, 4.00) | 1.00 (0.00, 4.00) | 1.00 (0.00, 5.00) | Z=-0.138 | 0.890 |
|  | 8 weeks | 0.00 (0.00, 3.00) | 1.00 (0.00, 3.00) | 0.00 (0.00, 3.00) | Z=-0.821 | 0.411 |
| WOMAC physical function score | baseline | 74.00 (60.00, 83.00) | 69.31 ± 21.61 | 76.00 (62.00, 83.00) | Z=-0.565 | 0.572 |
|  | 2 weeks | 60.00 (46.00, 72.00) | 54.09 ± 21.12 | 60.00 (52.00, 75.00) | Z=-1.843 | 0.065 |
|  | 4 weeks | 45.00 (41.00, 58.00) | 44.66 ± 18.53 | 49.53 ± 15.96 | t=-1.666 | 0.098 |
|  | 8 weeks | 34.00 (26.00, 43.00) | 32.00 (23.00, 39.00) | 39.07 ± 16.45 | Z=-2.622 | 0.009 |
| WOMAC total score | baseline | 100.00 (82.00, 111.00) | 94.37 ± 28.64 | 102.00 (89.00, 110.00) | Z=-0.604 | 0.546 |
|  | 2 weeks | 81.00 (66.00, 95.00) | 74.30 ± 28.15 | 85.00 (72.00, 101.00) | Z=-1.974 | 0.048 |
|  | 4 weeks | 64.00 (54.00, 78.00) | 61.29 ± 24.38 | 67.40 ± 21.23 | t=-1.581 | 0.116 |
|  | 8 weeks | 46.00 (34.00, 60.00) | 44.00 (31.00, 55.00) | 53.31 ± 22.47 | Z=-2.499 | 0.012 |

# Supplementary Material 10 Comparison of EuroQol5D-3L and EuroQol VAS between two groups at different time points

| Variable | Time | Total  (n = 140) | Osteoking Group  (n = 70) | NSAIDs Group  (n = 70) | Statistic | P |
| --- | --- | --- | --- | --- | --- | --- |
| EuroQol5D-3L | baseline | 0.54 (0.40, 0.54) | 0.54 (0.40, 0.54) | 0.54 (0.40, 0.54) | Z=-1.467 | 0.142 |
|  | 2 weeks | 0.54 (0.46, 0.63) | 0.54 (0.54, 0.63) | 0.46 (0.37, 0.46) | Z=-7.779 | <0.001 |
|  | 4 weeks | 0.63 (0.54, 0.81) | 0.63 (0.54, 0.83) | 0.63 (0.54, 0.73) | Z=-0.886 | 0.376 |
|  | 8 weeks | 0.81 (0.63, 0.91) | 0.91 (0.73, 0.91) | 0.73 (0.63, 0.83) | Z=-4.390 | <0.001 |
| EuroQol VAS | baseline | 45.00 (44.00, 60.00) | 50.00 (41.00, 60.00) | 45.00 (45.00, 60.00) | Z=-0.080 | 0.936 |
|  | 2 weeks | 60.00 (55.00, 70.00) | 61.74 ± 12.51 | 58.00 (51.00, 70.00) | Z=-1.457 | 0.145 |
|  | 4 weeks | 70.00 (65.00, 79.00) | 70.00 (65.00, 80.00) | 70.00 (60.00, 75.00) | Z=-2.393 | 0.017 |
|  | 8 weeks | 80.00 (70.00, 85.00) | 80.00 (79.00, 90.00) | 80.00 (70.00, 84.00) | Z=-2.671 | 0.008 |

# Supplementary Material 11 Incidence of adverse events (including adverse reactions) in the two groups

| serial number | HosPital Number | Group | Subject number | adverse events | PreferredTerms | System OrganClasses | Start time | End time | Severity level | Serious Adverse Event(Yes/No) | transition | Measures taken for research drugs | Whether to apply corrective treatment | Whether to withdraw from the test | Relationship between AE and test drug | Adverse Drug Reaction |
| --- | --- | --- | --- | --- | --- | --- | --- | --- | --- | --- | --- | --- | --- | --- | --- | --- |
| 1 | 1 | Osteoking Group | 103014 | Tiredness | Tiredness | Neuropathy | 2021-03-02 | 2021-03-14 | Mild | No | Disappeared | continued use | medication | No | related | Yes |
| 2 | 5 | Osteoking Group | 501004 | Dizziness | Dizziness | Heart-related diseases, symptoms and signs ( not otherwise classified ) | 2020-07-03 | 2020-07-03 | Mild | No | Disappeared | continued use | No intervention | No | related | Yes |
| 3 | 5 | Osteoking Group | 501006 | Throat dry | Dry throat | Respiratory, thoracic and mediastinal diseases | 2020-07-10 | 2020-07-10 | Mild | No | Disappeared | continued use | No intervention | No | related | Yes |
| 4 | 5 | Osteoking Group | 501012 | Dry throat | Dry throat | Respiratory, thoracic and mediastinal diseases | 2020-07-28 | 2020-07-28 | Mild | No | Disappeared | continued use | No intervention | No | related | Yes |
| 5 | 5 | Osteoking Group | 501020 | Dizziness | Dizziness | Heart-related diseases, symptoms and signs ( not otherwise classified ) | 2020-09-11 | 2020-09-11 | Mild | No | Disappeared | continued use | No intervention | No | related | Yes |
| 6 | 11 | Osteoking Group | 1101029 | Chest tightness | Chest discomfort | Heart organ disease | 2021-06-09 | 2021-06-11 | Mild | No | Mitigation | continued use | medication | No | unrelated | No |
| 7 | 13 | Osteoking Group | 1310053 | Toothache | Toothache | Gastrointestinal system diseases | 2021-05-26 | 2021-05-31 | Mild | No | Mitigation | continued use | medication | No | unrelated | No |
| 8 | 18 | Osteoking Group | 1805002 | Itch on the root of the left thigh | Pruritus | Skin and subcutaneous tissue diseases | 2020-09-29 | 2020-10-02 | Mild | No | Disappeared | continued use | No intervention | No | unrelated | No |
| 9 | 18 | Osteoking Group | 1805003 | Colds | Nasopharyngitis | Infection and infectious diseases | 2021-02-10 | 2021-02-13 | Mild | No | Disappeared | continued use | medication | No | unrelated | No |
| 10 | 18 | Osteoking Group | 1805006 | Colds | Nasopharyngitis | Infection and infectious diseases | 2021-04-20 | 2021-04-21 | Mild | No | Disappeared | continued use | No intervention | No | unrelated | No |
| 11 | 19 | Osteoking Group | 1902006 | Colds | Upper respiratory tract infection | Respiratory, thoracic and mediastinal diseases | 2020-07-20 | 2020-07-26 | Mild | No | Disappeared | continued use | medication | No | unrelated | No |
| 12 | 28 | Osteoking Group | 2801029 | Itchy throat discomfort | Throat stimulation | Respiratory, thoracic and mediastinal diseases | 2020-11-10 | 2020-11-12 | moderate | No | Disappeared | continued use | medication | No | unrelated | No |
| 13 | 29 | NSAIDs Group | 2906005 | Waist sprain | Ligament sprain | All kinds of damage | 2020-08-26 | 2020-09-03 | Mild | No | Disappeared | continued use | medication | No | unrelated | No |
| 14 | 29 | Osteoking Group | 2906007 | Cough | Cough | Respiratory, thoracic and mediastinal diseases | 2020-12-10 | 2020-12-25 | moderate | No | Disappeared | continued use | medication | No | unrelated | No |

# Supplementary Material 12 Toxicological Study of Osteoking

**Topic Name: 26-Week Repeated Toxicity Test of Henggu Bone Injury Healing Agent Administered via Rat Gavage**

Topic Code: W/ZY-18006-1B

Subject name: Permanent bone injury healing agent, permanent bone injury healing agent (5 times), permanent bone injury healing agent (10 times)

Subject Number: 18006(1), 18006(3), 18006(4)

Principal Investigator: Mao Yong

Commissioned by: Yunnan Kress Pharmaceutical Co., LTD

Research Institution: Drug Safety Evaluation Center of Yunnan Institute of Materia Medica

Research Start and End Date: November 30, 2018 - December 3, 2019

Archive Storage Location: Archives Room of Drug Safety Evaluation Center of Yunnan Institute of Materia Medica

Completion Date: December 3, 2019

Drug Safety Evaluation Center of Yunnan Institute of Materia Medica

Abstract This study utilized Sprague-Dawley (SD) rats to conduct a 26-week repeated toxicity test via gavage administration of Henggu Bone Injury Healing Agent with batch number 20181108, as well as its 5-fold and 10-fold concentrated formulations (batch numbers 20181109 and 20181111, respectively). After drug administration, a 5-week recovery observation period was implemented to observe the toxicity response and severity following repeated exposure to the test substances. The aim of this study was to provide experimental evidence for the clinical safety of the test substances.

224 SD rats were randomly divided into 4 groups based on gender and weight, with 28 males and 28 females in each group. These groups were: Control group (given 0.04% ethylparaben aqueous solution, referred to as the excipient group),Low-dose group of Henggu Gushang Healing Agent (raw drug 4.76g·kg-1, given Henggu Gushang Healing Agent), Medium-dose group (raw drug 23.08g·kg-1, given 5 times concentrated Henggu Gushang Healing Agent), High-dose group (raw drug 47.60g·kg-1, given 10 times concentrated Henggu Gushang Healing Agent). These three dose groups are referred to as low, medium, and high dose groups, respectively. They correspond to 46.6, 226.1, and 466.2 times the clinical dose for a six-year-old child (raw drug 0.1021g·kg-1), which translates to 7.0, 34.0, and 70.2 times the equivalent clinical dose for a six-year-old rat (raw drug 0.6782g·kg-1). They also correspond to 93.2, 451.7, and 931 times the clinical dose for a twelve-year-old child (raw drug 0.0511g·kg-1), which translates to 18.7, 90.8, and 187.3 times the equivalent clinical dose for a twelve-year-old rat (raw drug 0.2542g·kg-1). Finally, they correspond to 67.2, 326.0, and 672.3 times the clinical dose for an adult (raw drug 0.0708g·kg-1), which translates to 14.6, 70.7, and 145.8 times the equivalent clinical dose for an adult rat (raw drug 0.3265g·kg-1).

During the experimental period, the food intake, water consumption, body weight, external signs, and behavioral activities of the animals in each group were observed. At the end of 4, 13, and 26 weeks of administration, 48, 48, and 79 animals (12 males and 12 females in each group, with 9 males in the high-dose group due to the death of one male rat at 23 weeks) were selected, fasted for 12h9min to 13h45min with water access allowed. After isoflurane anesthesia, blood was collected from the abdominal aorta for hematological, serum biochemical, and electrolyte analysis. The animals were dissected, and sternal bone marrow smears were prepared for further examination. Macroscopic observations were made of the organs and tissues, tibia lengths were measured, organ weights were taken, and organ coefficients were calculated. Histopathological examinations were performed on animals in the excipient group and the high-dose group. The remaining 48 animals (12 males and 12 females in each group) were observed for 5 weeks after drug withdrawal, and the same tests were performed as those at the end of 26 weeks of administration. The results showed:

General symptoms and signs

During the experimental period, all animals in the excipient control group and the low-dose group exhibited shiny fur, normal behavior, and good mental status without any abnormal general conditions.

During the administration period, only one female rat in the medium-dose group showed increased activity, excitement, and jumping intermittently or continuously for 6 days between weeks 13 and 16, lasting from 14 to 47 minutes before recovery. In the high-dose group, only one female rat showed a single episode of brownish-soft stool during week 19 (for 1 day). Two female rats displayed intermittent convulsive seizures for a total of 7 days during weeks 12-15, 18, and 25, lasting for 1 minute before recovery. Among 14 male rats from weeks 19 to 26 (a total of 36 days), single, intermittent, or continuous episodes of brownish-soft stool were observed. Among them, 2-3 male rats also exhibited brownish-loose stool and/or uncleanliness around the anus. Additionally, from the 4th week to the end of 26 weeks of administration, both male and female rats showed an increased amount of feces when they were transferred to new cages.

Among them, while a very few female rats in the medium-dose group (1♀) and some female rats in the high-dose group (2♀) intermittently displayed symptoms similar to those of central nervous system and/or somatic motor dysfunction, the duration of these symptoms was short, and the frequency of occurrence in both groups was low and relatively equivalent. These symptoms were not observed in female rats from the low-dose group or male rats from any of the three dose groups, indicating no dose-response relationship or trend. Therefore, it is believed that the aforementioned symptoms observed in a few female rats in the medium and high-dose groups are likely related to individual differences and sensitivities of the animals themselves, and are not significantly correlated with the test substance. The soft stool observed in a very few female rats and half of the male rats in the high-dose group, as well as the toxic reactions of loose stool and uncleanliness around the anus observed in some male rats, were slightly more prominent in male rats compared to female rats. There was a certain dose-response trend, and the number, type, and frequency of these symptoms gradually increased in male rats over time. Therefore, it is considered that the autonomic nervous system and gastrointestinal reactions observed in male rats in the high-dose group are somewhat correlated with the test substance, and these reactions tend to increase over time with continuous administration.

Feeding evaluation

During the administration period, the average food intake of male rats in the low-dose group was comparable to that of the excipient control group during week 15. However, the average food intake of female and male rats in the low, medium, and high-dose groups was slightly lower than that of the excipient control group. Except for weeks 14, 17, and 18 for female rats and weeks 16, 19, 21, and 25 for male rats, there was a dose-response relationship in the changes in food intake among the three dose groups for both sexes. The decrease in food intake was slightly greater and lasted longer in female rats compared to male rats, with a more prominent effect in the medium-dose group and a more significant effect in the high-dose group. This may be closely related to the physicochemical and biological properties of the test substance, the concentration of the drug solution, the large administration volume, and the twice-daily dosing regimen for 26 consecutive weeks, which affected the appetite of the animals. There was no clear correlation with the toxic reaction of the test substance itself. During the recovery period after drug withdrawal, the average food intake of the treatment groups was slightly higher in male rats compared to the excipient control group, while it remained slightly lower in female rats. However, the average food intake of female and male rats in the low, medium, and high-dose groups showed varying degrees of recovery.

Water Consumption Evaluation

During the administration period, the average water consumption of female rats in the low-dose group during weeks 1, 2, 14, 18, 20, 21, and 24 to 26, and male rats during weeks 1 to 3, 5, 9, 11, 12, 13, 16, 17, 19 to 25, was slightly higher than that of the excipient control group. In the medium-dose group, the average water consumption of female rats during weeks 1 to 4 and 14 to 21, and male rats during weeks 1 to 5 and 7 to 26, was also higher. For the high-dose group, the average water consumption of both sexes was higher during weeks 1 to 26 (except for week 13 for female rats). There was a dose-response trend in water consumption during weeks 1 to 4 and 14 to 21 for female rats and weeks 2, 12, 13, 17 to 21, and 23 to 26 for male rats.

During the recovery period, the average water consumption of male and female rats in all three dose groups gradually declined with time, returning to slightly below (for females) and slightly above (for males) the levels of the excipient control group by the fifth week of recovery. No dose-response trend or statistical difference was observed.

In summary, the administration of the test substance increased the water consumption of the animals, with a more pronounced effect in the medium-dose group and a more significant effect in the high-dose group. The increase was slightly more prominent in male rats compared to female rats. This increase is primarily attributed to the clinical observation of dry mouth after taking the test substance, leading to a corresponding increase in water demand. It may also be related to the physicochemical and biological properties of the test substance, the concentration of the drug solution, and the increased fecal volume and/or soft stool observed in the high-dose group, which can lead to excessive fluid loss and compensatory water intake by the animals.

Body Weight Evaluation

During the administration period, the average body weight of male and female rats in the low, medium, and high-dose groups generally increased, with the exception of slightly slower weight gain in the high-dose group during certain weeks. The average body weight of female and male rats in the low and high-dose groups, as well as male rats in the medium-dose group, was slightly lower than that of the excipient control group, but no statistical differences were observed among the groups. There was a dose-response trend only in the change in body weight of male rats during weeks 1 to 3.

During the recovery period, the average body weight of male and female rats in all three dose groups recovered to some extent, and no differences were observed among the groups. The magnitude of the decrease in body weight was also gradually diminishing in male rats from the low-dose group.

In conclusion, the administration of the test substance led to a slower increase in body weight among male and female rats, with a slightly more prominent effect in male rats. This may be related to the physicochemical and biological properties of the test substance, the concentration of the drug solution, as well as the presence of soft stool and/or increased stool volume, which may have reduced food intake and consequently led to a relatively slower increase in body weight.

Hematological Indices Evaluation

① After 4 weeks of administration, the mean corpuscular volume (MCV), platelet count (PLT) in female rats, and mean corpuscular hemoglobin concentration (MCHC) in male rats of the low, medium, and high-dose groups were slightly higher than those of the excipient control group. The reticulocyte count (#Retic) and reticulocyte percentage (Retic%) in female rats of the medium and high-dose groups were also slightly elevated. In contrast, the red blood cell count (RBC), #Retic, thrombin time (TT) in male rats of all three dose groups, and RBC in female rats of the high-dose group, were slightly lower than the control group. Among these, the hemoglobin (HGB), hematocrit (HCT), and RBC showed a similar decreasing trend in female rats of the high-dose group and male rats of the medium and high-dose groups. A dose-response trend was observed for most parameters, except #Retic, RBC, and MCV, but only MCV showed a statistical difference between groups.

② After 13 weeks, the red cell distribution width (RDW), monocyte percentage (MONO%), fibrinogen (FIB), Retic%, and #Retic in female rats, as well as neutrophil percentage (NEU%) and PLT in male rats of all three dose groups, were slightly higher than the control group. The plateletcrit (PIT) in female rats of the low and medium-dose groups was also elevated. Meanwhile, the mean corpuscular hemoglobin (MCH) and TT in male rats of all three dose groups were lower, while RBC in both sexes of the high-dose group was lower than the other groups. Again, HGB, HCT, and RBC displayed a similar decreasing trend in both sexes of the high-dose group. A dose-response trend was observed for most parameters, except PLT, TT, and RBC, but only RDW, RBC, Retic%, #Retic, and TT showed statistical differences between groups.

③ After 26 weeks, the HGB and HCT in female rats, as well as basophil percentage (BASO%), RBC, HGB, and HCT in male rats of all three dose groups, were slightly lower than the control group. The RDW in male rats of the low-dose group was also lower. In contrast, the prothrombin time (PT) in male rats and PLT, #Retic, and Retic% in both sexes of the medium and high-dose groups were higher. There was a similar decreasing trend in RBC, HGB, and HCT in female rats of the medium and high-dose groups. A dose-response trend was observed for most parameters, except RDW, PLT, #Retic, and Retic%, but only RDW, #Retic, and Retic% showed statistical differences between groups.

④ After 5 weeks of recovery, the RDW in female rats and MONO% in male rats of all three dose groups were slightly higher than the control group, showing a dose-response trend. However, no statistical differences were observed between groups.

Tip:  RBC, HGB and HCT decreased to different degrees and PLT, #Retic and Retic% increased to different degrees in both male and female rats at different detection time points after administration (more significant at medium dose and more significant at high dose).  This may be related to the slight fluctuation of the above indexes indirectly caused by the adverse reactions such as gastrointestinal reactions in the animals, partial loss of body fluids, partial compensatory hydration and reduced feeding.  The irregular microwave movement of coagulation indexes, including the decrease of TT at individual detection time points of male mice, and the increase of FIB and PT at individual detection time points of female mice, may be related to the slight influence of the test subject on activating blood and benefiting qi, but the changes were not obvious.  There was no clear correlation between the fluctuation of the above indexes and the given subjects.

Serum Biochemistry and Electrolyte Tests

① After 4 weeks of administration, only female rats in the low, medium, and high-dose groups showed decreases in ALP, GGT, BUN, CREA, and GLDH, while GLU increased in the low and high-dose groups, and TG increased in the high-dose group. Except for GGT, there were differences between groups, but only GGT showed a dose-response trend. The increases in GLU and TG were mainly due to high values in individual animals and were not significantly related to the test substance. The decreases in the other indices were not of significant toxicological importance.

② After 13 weeks of administration, female rats in all dose groups showed decreases in ALT, CREA, Cl-, and CHE, while male rats showed decreases in CHE, and males in the high-dose group showed a decrease in Na+. There was a dose-response trend only in female rats' ALT, CREA, Cl-, and male rats' CHE. Only Na+ showed a statistical difference between groups. The decreases in ALT, CREA, and CHE were not of significant toxicological importance, and no obvious abnormalities were observed in the heart weight, coefficient, or histopathology of the heart tissue of both sexes. Therefore, the increases in CK, CKMB, and LDH in both sexes were not considered to be definitively related to the test substance. The decreases in Cl- and Na+ in the medium and high-dose groups may be related to increased water intake and gastrointestinal reactions in the high-dose group. The slightly more significant increase in PINP in female rats was not due to high values in individual animals, so it is speculated that there may be a certain correlation with the test substance increasing bone formation markers.

③ After 26 weeks of administration, female rats in all dose groups showed decreases in AST, ALT, CHOL, Na+, GLDH, and C3, while male rats showed decreases in ALT, CHOL, LDH, GLDH, PINP, and IgG. Female rats showed increases in GGT, E2, and 5-HT. Except for female rats' GLDH and male rats' IgG, there was a dose-response trend, but only female rats' AST, ALT, GLDH, E2, and male rats' CHOL, GLDH, IgG showed statistical differences between groups. The decreases in AST, ALT, GLDH in female rats and ALT, LDH, GLDH, PINP, IgG in male rats were not of significant toxicological importance. The slight decreases in C3 and increases in GGT in female rats were not significant. The decreases in CHOL in both sexes and Na+ in female rats may be related to reduced food intake and gastrointestinal reactions in the high-dose group. The increases in E2 and 5-HT in female rats were not due to high values in individual animals, so it is speculated that they may be related to the test substance increasing sex hormone and neurotransmitter levels, which promote bone formation.

④ After 5 weeks of recovery, female rats in all dose groups showed increases in ALP, Na+, and Cl-, while male rats showed increases in ALP and BUN. Male rats showed decreases in AST, GLDH, and IgG. Except for Na+ and Cl-, there was a dose-response trend, but only Na+ and Cl- showed statistical differences between groups. The increases in ALP and BUN were minimal, and the decreases in AST, GLDH, and IgG were not of significant toxicological importance. Therefore, after 5 weeks of recovery, there were no significant abnormalities in the relevant indicators of the animals.

Organ Weights and Indices

In the low, medium, and high-dose groups, the liver weights, liver-to-body weight ratios, and liver-to-brain weight ratios of female rats after 4 and 13 weeks of administration, as well as male rats after 26 weeks of administration, were found to be higher than those in the excipient control group to varying degrees. Comparisons between groups showed differences and a certain dose-response trend or relationship. Additionally, the liver weights and liver-to-body weight ratios of male rats in the medium-dose group after 4 weeks, the liver weights of male rats in the high-dose group after 13 weeks, female rats in the medium-dose group after 26 weeks, and the liver-to-body weight ratios of male rats in the medium and high-dose groups after 13 weeks and female rats after 26 weeks were also higher than those in the excipient control group, but there were no significant differences or dose-response trends when compared between groups.

After 5 weeks of recovery, the liver weights, liver-to-body weight ratios, liver-to-brain weight ratios of female rats in the high-dose group, and the liver weights and liver-to-body weight ratios of male rats in all dose groups were still slightly higher than those in the excipient control group, but there was no dose-response trend. Only the differences in liver-to-body weight ratios between female rat groups were significant. Although no significant abnormal changes in serum biochemistry and histopathological examination of the liver related to the test substance were observed in both sexes, it cannot be excluded that the varying degrees of increase in liver weights, liver-to-body weight ratios, and liver-to-brain weight ratios after administration at different time points in both sexes are unrelated to the test substance. After 5 weeks of discontinuation, the extent of these changes decreased, showing a trend of recovery.

After 4 weeks of administration, the left kidney weights, left kidney-to-body weight ratios, right kidney weights, right kidney-to-body weight ratios, and total kidney weights and kidney-to-body weight ratios in female rats from the low, medium, and high-dose groups, as well as the right kidney weights and kidney-to-body weight ratios in the high-dose group, were slightly higher than those in the excipient control group. Among them, the kidney-to-body weight ratios of the left and right kidneys in male rats showed a dose-response trend, but only the differences in the left kidney-to-body weight ratios between groups were significant. No differences or dose-response trends were observed in the kidney-to-brain weight ratios.

After 13 and 26 weeks of administration, the left kidney weights, left kidney-to-body weight ratios, right kidney weights, right kidney-to-body weight ratios, total kidney weights, kidney-to-body weight ratios, and kidney-to-brain weight ratios in male rats from all dose groups were slightly higher than those in the excipient control group. Comparisons between groups showed significant differences and dose-response trends or relationships. In female rats after 13 weeks of administration, the left kidney weights, left kidney-to-body weight ratios, right kidney weights, right kidney-to-body weight ratios, and total kidney weights and kidney-to-body weight ratios were slightly higher than those in the excipient control group, showing a dose-response trend, but there were no significant differences between groups. After 26 weeks of administration in female rats, the left kidney weights, left kidney-to-body weight ratios, right kidney weights, right kidney-to-body weight ratios, total kidney weights, kidney-to-body weight ratios, and kidney-to-brain weight ratios in the medium and high-dose groups were slightly higher than those in the excipient control group and slightly higher than those in the low-dose group. Except for the left kidney-to-body weight ratio, there were significant differences between groups, but no dose-response trend was observed.

After 5 weeks of recovery, the right kidney weights, kidney weights, and kidney-to-body weight ratios of female rats in the high-dose group, as well as the left kidney weights, left kidney-to-body weight ratios, right kidney weights, right kidney-to-body weight ratios, total kidney weights, kidney-to-body weight ratios, and kidney-to-brain weight ratios of male rats in all dose groups, were still slightly higher than those in the excipient control group. There were still differences between groups, but no dose-response trends or relationships were observed.

Although no abnormal changes in serum biochemistry and histopathological examination of the kidneys related to the test substance were observed in both sexes, considering the significant increase in water intake during drug administration, which may indirectly increase the burden on the kidneys and subsequently lead to an increase in kidney weights, it cannot be excluded that the increase in kidney weights and kidney-to-body weight ratios in both sexes at different time points after administration is indirectly or possibly directly related to the test substance. After 5 weeks of discontinuation, the extent of these changes decreased, showing a trend of recovery.

Systematic Anatomy Gross Examination and Histopathological Analysis

Grossly, during the early stage of drug administration, one male rat in the medium-dose group did not show atrophy of the right testis or right epididymis. In the intermediate stage, uterine distension was observed in one animal each from the low- and high-dose groups. By the end of the administration period, uterine distension was noted in one animal from the low-dose group and two animals from the high-dose group. During the recovery phase, uterine distension was observed in one animal each from the excipient control group, low-dose group, and high-dose group, and two animals from the medium-dose group. These changes are likely to be occasional abnormalities due to individual differences in physiological cycles or growth and development during the animals' life cycle, and are unrelated to the test substance. Other than the aforementioned observations, the shape, size, color, and texture of the organs and tissues in all other animals, including those that died during the high-dose group, were normal.

Microscopically, during the early, intermediate, and late stages of drug administration, small focal necrosis of hepatocytes in the liver and interstitial inflammatory cell infiltration in the prostate were observed in the high-dose group. During the recovery phase, interstitial inflammatory cell infiltration in the prostate was also noted in the high-dose group, and small focal necrosis of myocardial cells was observed in some animals. The severity of these lesions was mild, and similar changes were also seen in the excipient control group. There were no significant differences in the severity or incidence of these lesions between the high-dose group and the excipient control group (P>0.05). Furthermore, these lesions are common in rats and are considered spontaneous changes unrelated to the test substance.

A male rat in the high-dose group died on the 5th day of the 23rd week of drug administration (i.e., the 159th day of administration). Upon gross examination, brownish secretions were observed around the nose and mouth, and a similar brownish fluid resembling the drug solution was visible after dissection and sampling of the lungs. Microscopic histopathological examination revealed the following: ①Pulmonary hemorrhage with homogeneous or flocculent red-stained material in the alveoli；②Loss of mucosal epithelium on the trachea and left and right main bronchi, hemorrhage and inflammatory cell infiltration in the lamina propria, and fibrinoid material in the lumen；③Hepatic congestion, which is commonly seen in animals that die prematurely；

In summary:

① During the initial four weeks of drug administration (corresponding to a human age range from six years to adolescence or near adulthood), various indicators showed that the test substance slightly reduced food intake, resulting in a slightly slower increase in body weight. This led to a compensatory increase in water consumption that was indirectly related to the test substance. Furthermore, it indirectly or directly caused irregular minor fluctuations in RBC, HGB, HCT, PLT, #Retic, and Retic%, as well as slight increases in liver and kidney weights and coefficients.

② When the animals continued to receive repeated drug administration for 13 weeks (corresponding to a human age range from six years to adulthood) or 26 weeks (corresponding to a human age range from six years to adulthood and beyond), the extent of the changes observed at the four-week mark increased slightly. Additionally, symptoms of autonomic nervous system and gastrointestinal dysfunction emerged, along with decreases in serum Na+, Cl-, and CHOL levels, and increases in PINP, E2, and 5-HT. Throughout the entire administration period, the adverse reactions/toxic effects of the test substance on animals at different stages showed a partial dose-response trend or relationship, but no clear time-response trend or relationship. The overall impact was relatively minor and gradually resolved after drug withdrawal.

③ At various stages of drug administration, there were no obvious abnormalities in the external appearance and fur of male and female rats. Serum TCa concentrations were largely equivalent. Only female rats showed an increase in E2 levels at the end of the treatment period, which was related to pharmacological effects. Tibia length gradually increased with age and showed no significant abnormalities. The morphology and number of various cell types in the sternum and femur, as well as the structure, thickness, and other pathological features of the bone cortex and trabeculae, were all normal. Pathological examinations of other major organs and tissues also showed no abnormalities. Therefore, it is believed that the test substance did not significantly affect the growth and development of the animals.

Suggestions: In the process of daily repeated drug use, the amount, frequency and frequency of each use should be strictly controlled in accordance with the drug instructions, and the combined or simultaneous use of drugs that can affect the central and autonomic nervous system and physical movement, as well as the liver, kidney and gastrointestinal tract should be avoided as far as possible;  If necessary, attention should be paid to gastrointestinal reactions, daily appetite, water intake, and weight changes, or to monitor changes in blood routine, blood lipids, electrolytes, and related indicators such as liver and kidney function.

Conclusion: Under the experimental conditions, the samples of Henggu Gushang Healing Agent with batch number 20181108 and its 5-fold and 10-fold concentrations (batch numbers 20181109 and 20181111) were administered to SD rats continuously for 26 weeks at three doses of crude drug 4.76, 23.08, and 47.60 g·kg-1, followed by a 5-week recovery period. The results indicate that: ① After repeated administration of the test substance, rats corresponding to the age range from six years to adolescence or near adulthood exhibited very minor adverse reactions, and the degree and type of adverse reactions increased slightly with prolonged drug use. However, rats corresponding to the age range from six years to adulthood and beyond did not show significant abnormalities in growth and development. After drug withdrawal, the adverse reactions gradually disappeared, and the degree of abnormal indicator changes gradually decreased. No serious/irreversible/delayed toxicity was observed in rats corresponding to different age groups for clinical use. ② The non-toxic dose is less than 4.76 g·kg-1 of crude drug. The safe clinical dosage ranges for six-year-olds, twelve-year-olds, and adults are below 46.6, 93.2, and 67.2 times their respective age-specific doses, equivalent to 7.0, 18.7, and 14.6 times the equivalent human clinical doses for six-year-olds, twelve-year-olds, and adults in rats.

# Supplementary Material 13 Pharmacological Study of Osteoking

Modern pharmacological research on the main components of drugs in SUXIAOGUSHANGYUHEJI

SUXIAOGUSHANGYUHEJI(SGH) is a new preparation developed over the past decade based on classical traditional medical theory and modern pharmaceutical theory for the treatment of bone injuries. Traditional medicine believes that this formula has the effects of nourishing the liver and kidney, nourishing qi and blood, promoting blood circulation and removing stasis, anti-inflammatory and anti-inflammatory effects, and promoting muscle and bone connection. According to modern pharmacological research results, SGH contains multiple active ingredients and has a wide range of pharmacological effects: (1) it can stimulate the myocardium, increase cardiac output, improve coronary perfusion, dilate blood vessels, reduce peripheral resistance, and enhance systemic blood circulation. (2) Inhibiting the synthesis of thromboxane A ₂ (TXA ₂) by inhibiting cyclooxygenase, lipoxygenase, and thromboxane A ₂ synthase; By activating adenosine cyclase and inhibiting phosphodiesterase, the content of cyclic adenosine monophosphate (cAMP) in platelet cells increases, inhibiting collagen and adenosine diphosphate (ADP) induced aggregation of platelets, thereby exerting a significant anti platelet aggregation and inhibitory effect on thrombus formation (i.e., producing blood). Some other active ingredients in SGH have inhibitory effects on the vascular motor center and can also block the adrenergic presynaptic membrane in the periphery α- R. Reduce the release of norepinephrine; Some components block Ca by specifically blocking the longitudinal calcium ion channels of vascular smooth muscles ² Inflow and intracellular Ca ² Release, thereby causing peripheral vascular dilation, has a particularly significant dilation effect on spasmodic small blood vessels caused by pathological factors, thereby improving microcirculation of tissues and organs, and improving blood and oxygen supply to damaged tissue cells. (3) Some active ingredients in SGH exert strong anti-inflammatory, anti-inflammatory, and analgesic effects by inhibiting the effects of hyaluronidase, proteolytic enzymes, chondroitinase, histamine, serotonin, prostaglandin synthase, and enhancing the pituitary adrenal cortex system. Pharmacological studies have shown that the active ingredients in SGH can significantly inhibit acute and chronic inflammation caused by various inflammatory substances such as cotton balls, plastic rings, egg white, carrageenan, 5-hydroxytryptamine, kaolin, xylene, formaldehyde, dextran, and croton 3. While significantly inhibiting vascular permeability and reducing inflammatory infiltration, they also activate the microcirculation of inflammatory tissues, accelerate blood flow, and exhibit significant anti-inflammatory and anti-inflammatory effects. (4) Among the active ingredients in SGH, there are many that have anti pathogen effects. Not only does it have antibacterial effects on various Gram positive and negative bacteria such as Staphylococcus aureus, Streptococcus hemolyticus, Pneumococcus diphtheriae, Pseudomonas aeruginosa, Escherichia coli, Pneumocystis, Bacillus subtilis, and Catanella, but it also has inhibitory effects on fungi such as Clostridium oxysporum, Clostridium ferrorrhoeae, and Nocardia stellate, as well as viruses such as influenza, showing a broad-spectrum anti pathogen effect. (5) SGH contains abundant amino acids and trace elements. Among the 17 amino acids contained, up to eight are required: threonine, valine, methionine, leucine, isoleucine, phenylalanine, lysine, and histidine. In addition, it also contains osteogen. There are more than ten types of trace elements such as calcium, phosphorus, iron, copper, zinc, strontium, magnesium, manganese, lanthanum, cesium, scandium, etc. The role of these amino acids and trace elements in bone injury repair cannot be ignored. (6) The strong and nourishing effects of traditional Chinese medicines such as ginseng, Panax notoginseng, and Astragalus membranaceus in SGH have been well known by scholars at home and abroad. They contain active ingredients that activate tissue cells, enhance the body's anti damage, anti hypoxia, anti fatigue, anti high temperature, low temperature and other effects, and also have central sedative and analgesic effects (known as "adaptation to the original" effect). The above-mentioned pharmacological effects of drugs in SGH create an extremely favorable internal and external environment for the healing of bone injuries, providing the necessary material basis for bone injury repair, thereby accelerating the healing of bone injuries and shortening the course of the disease. In our pharmacological experiments, unique therapeutic effects were demonstrated. In the treatment of pathological models of fractures of the same type, it is the fastest formula for bone healing.

# Supplementary Material 14 Stability Study of Osteoking

Yunnan Kress Pharmaceutical Co., Ltd HENGGUGUSHANGYUHEJI Continuous stability assessment report


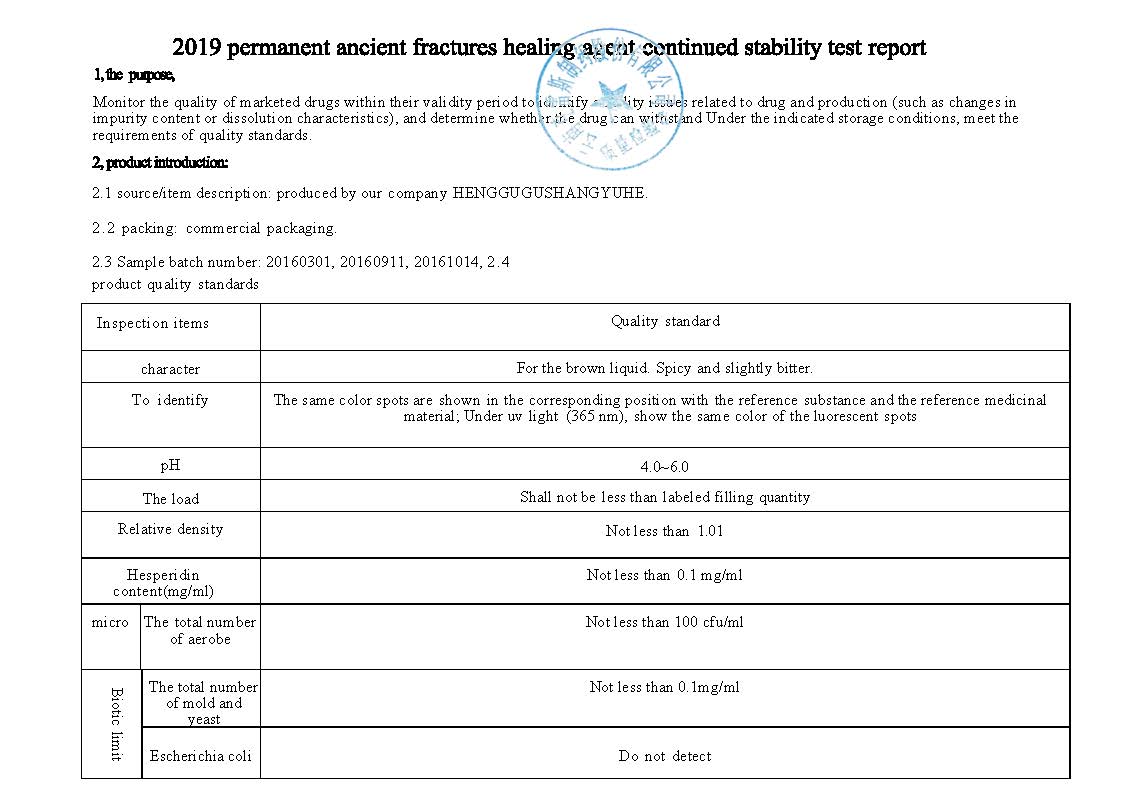


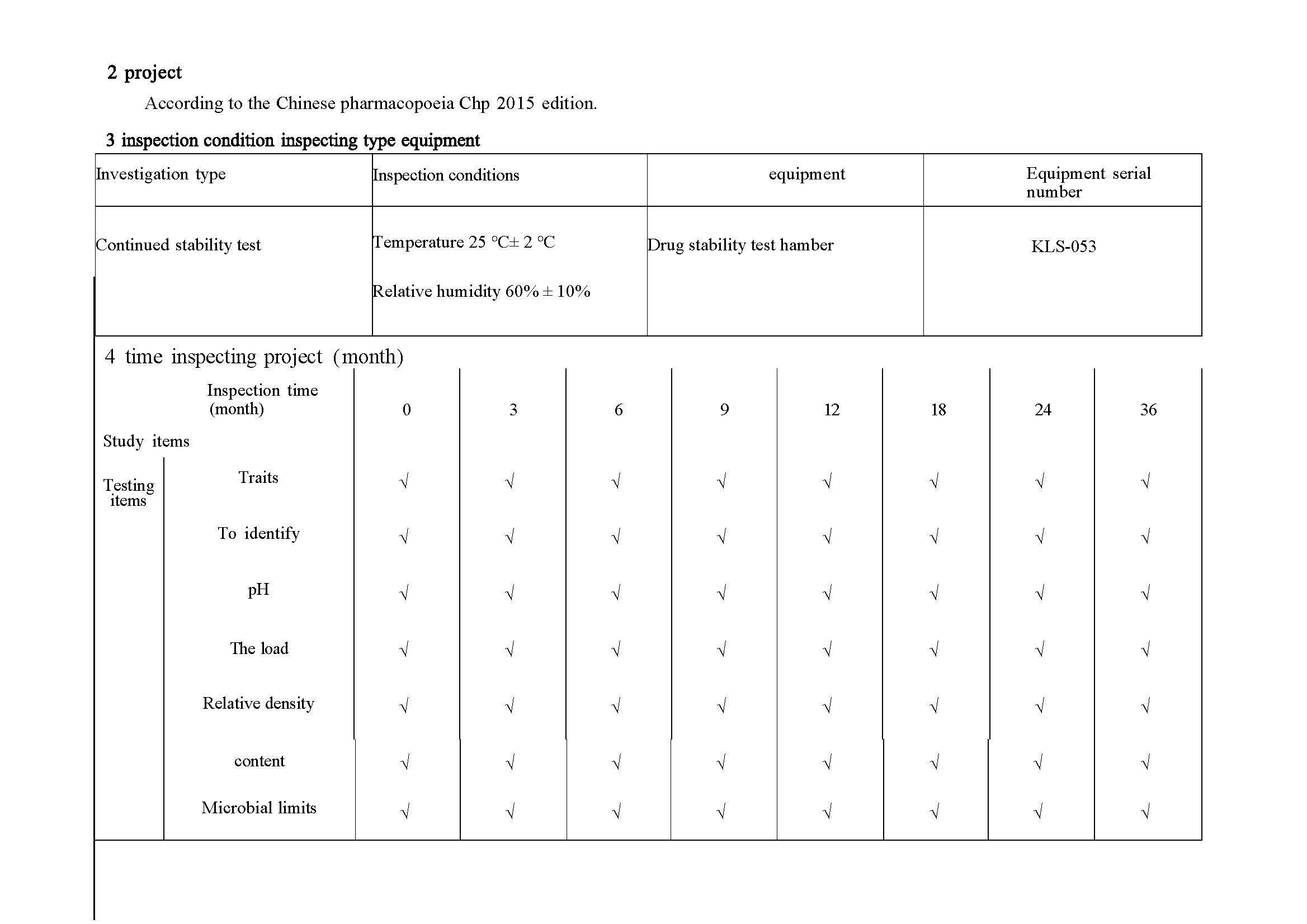


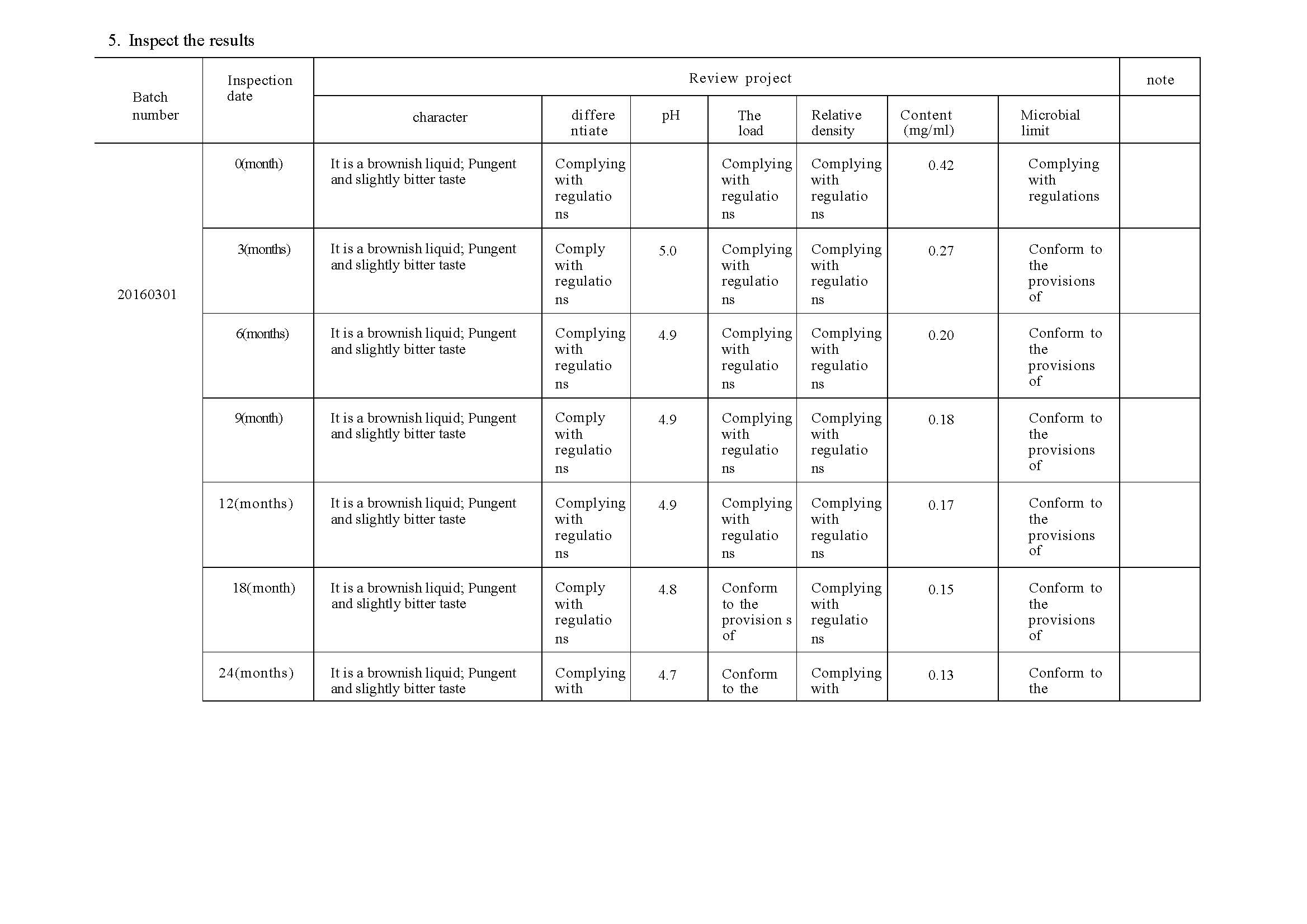


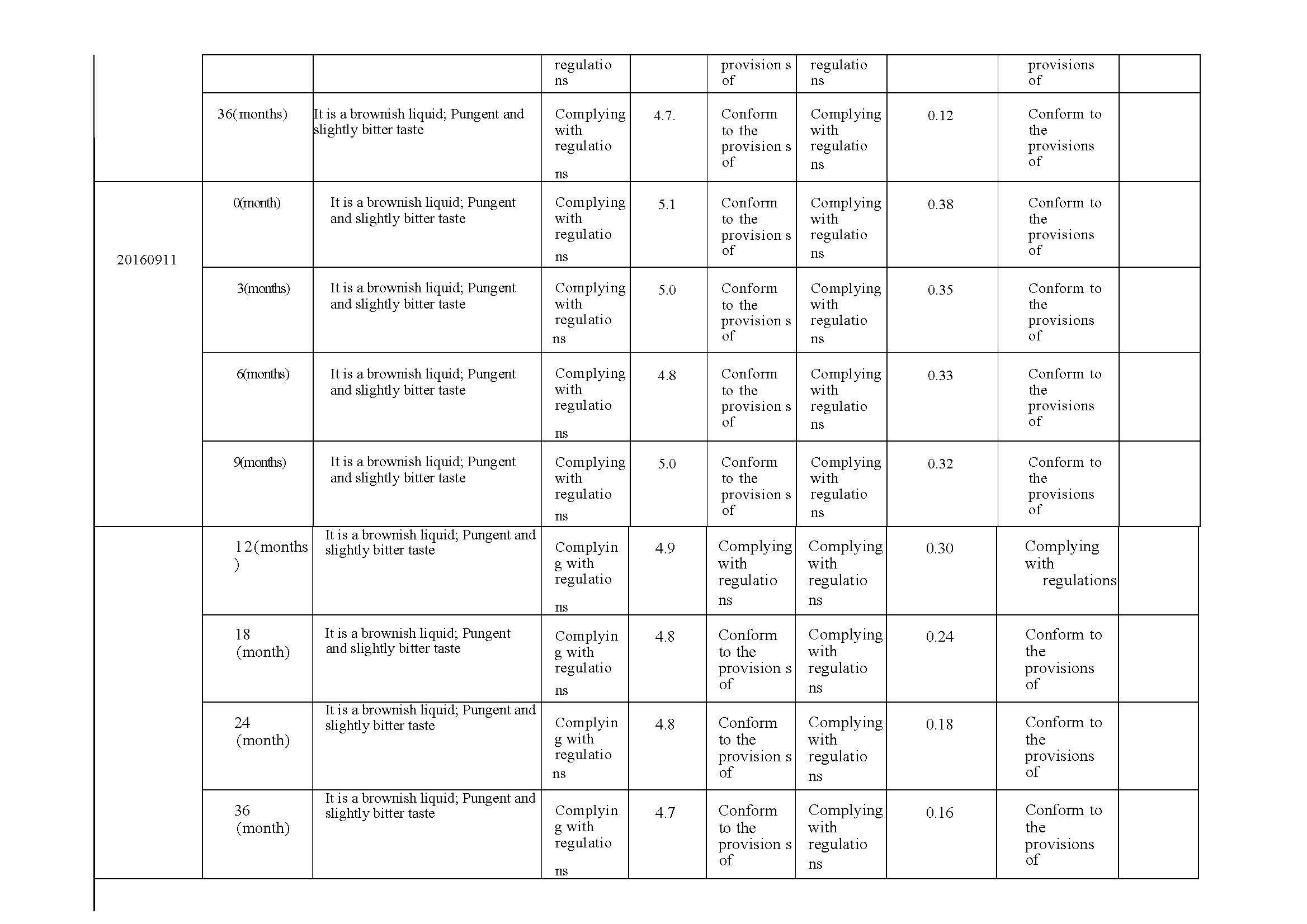


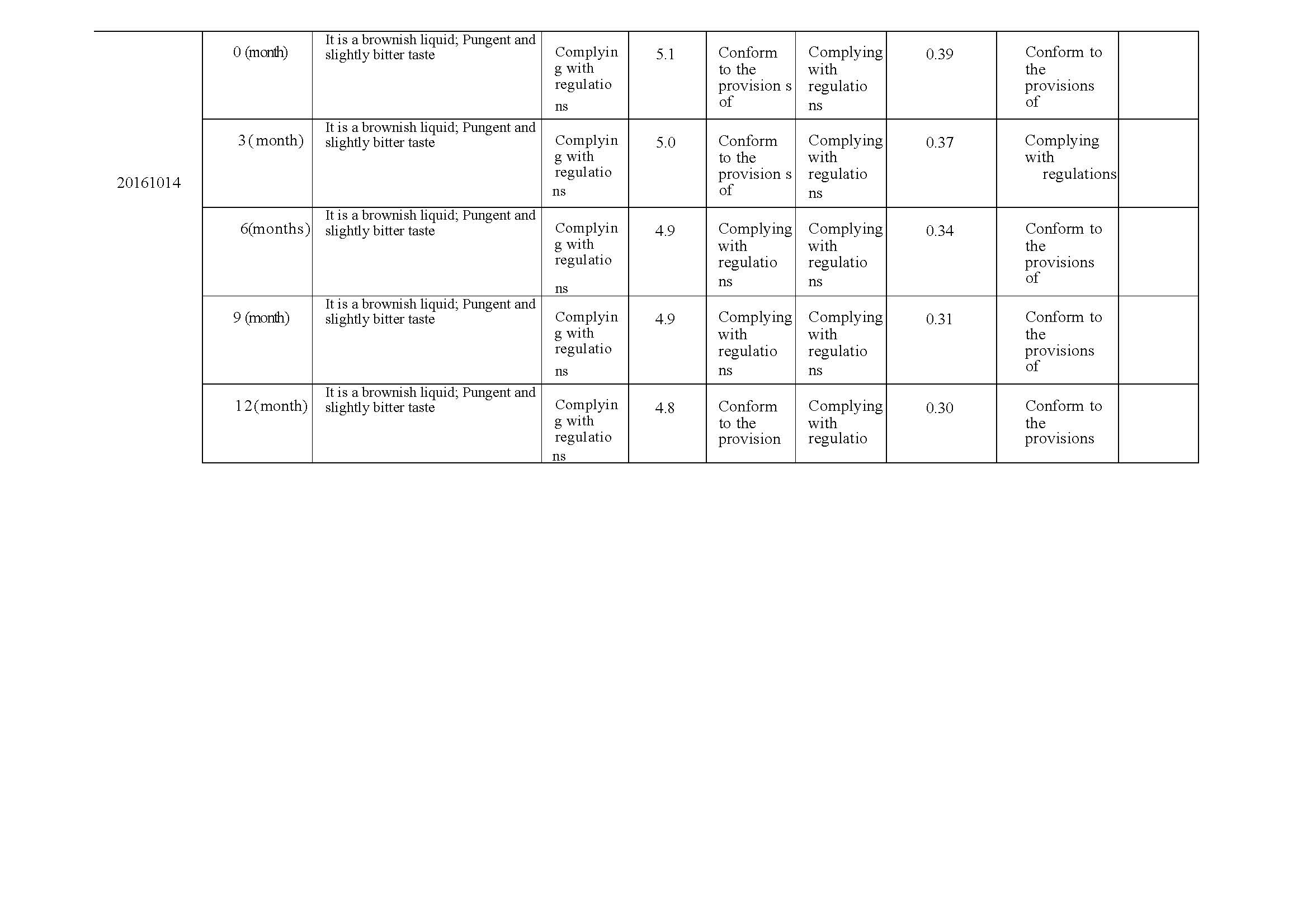


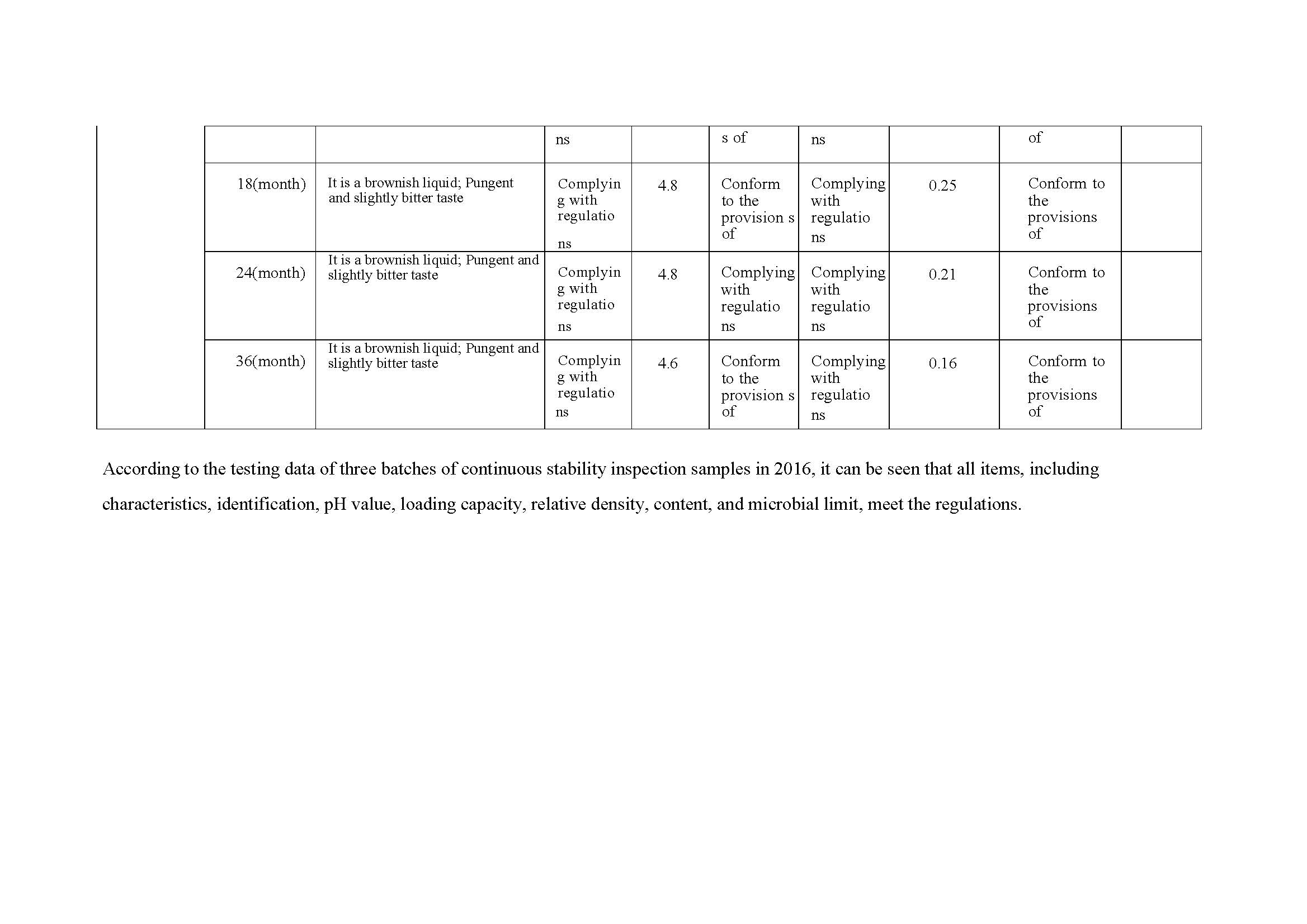


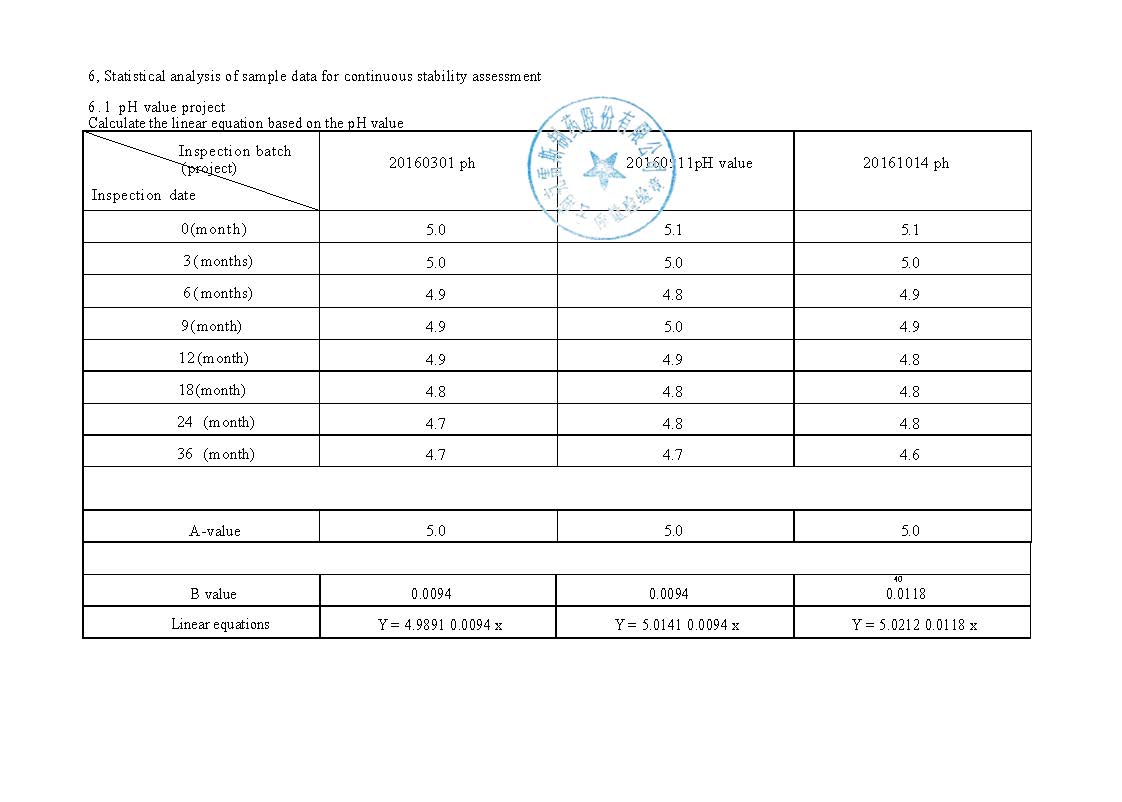


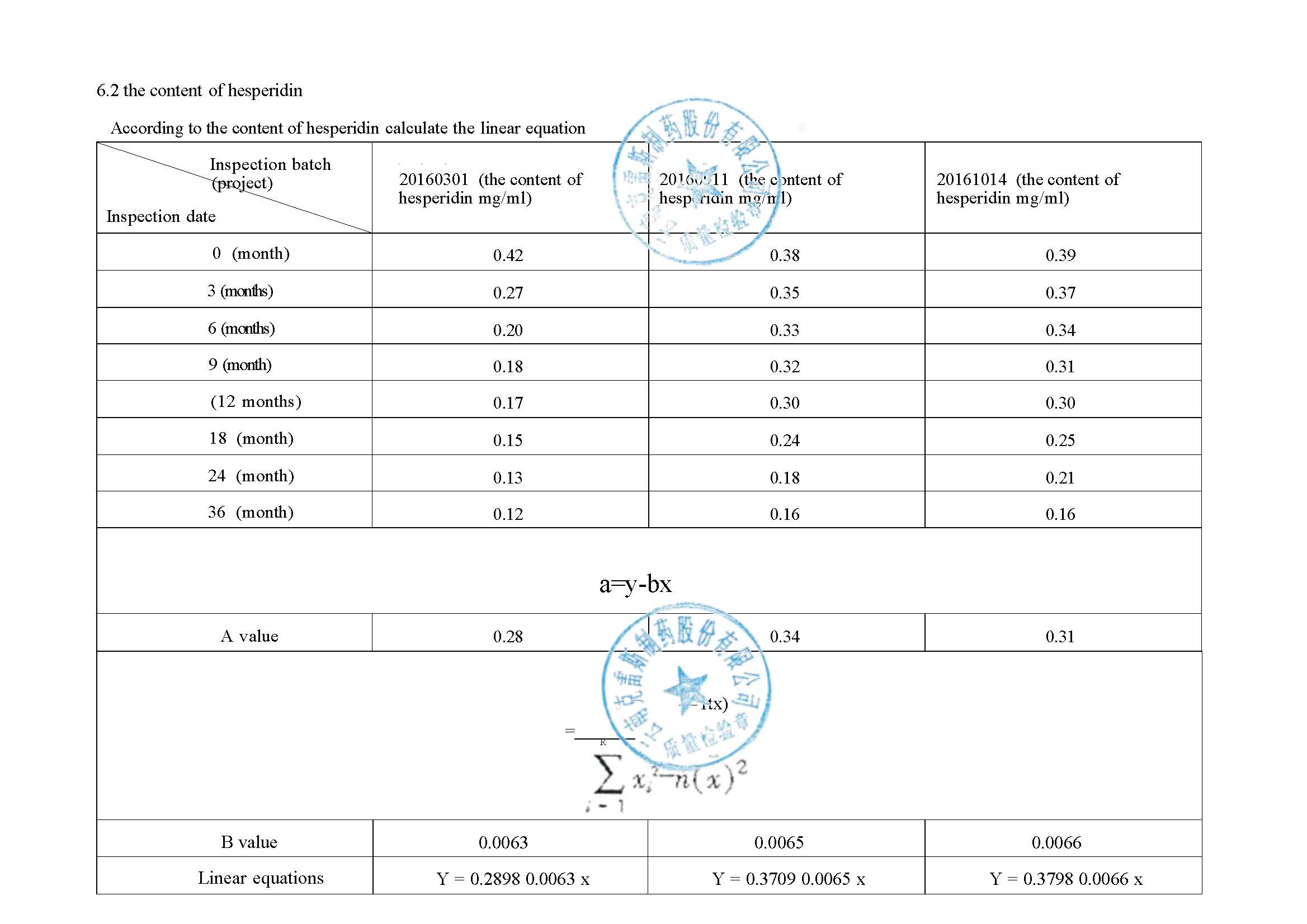


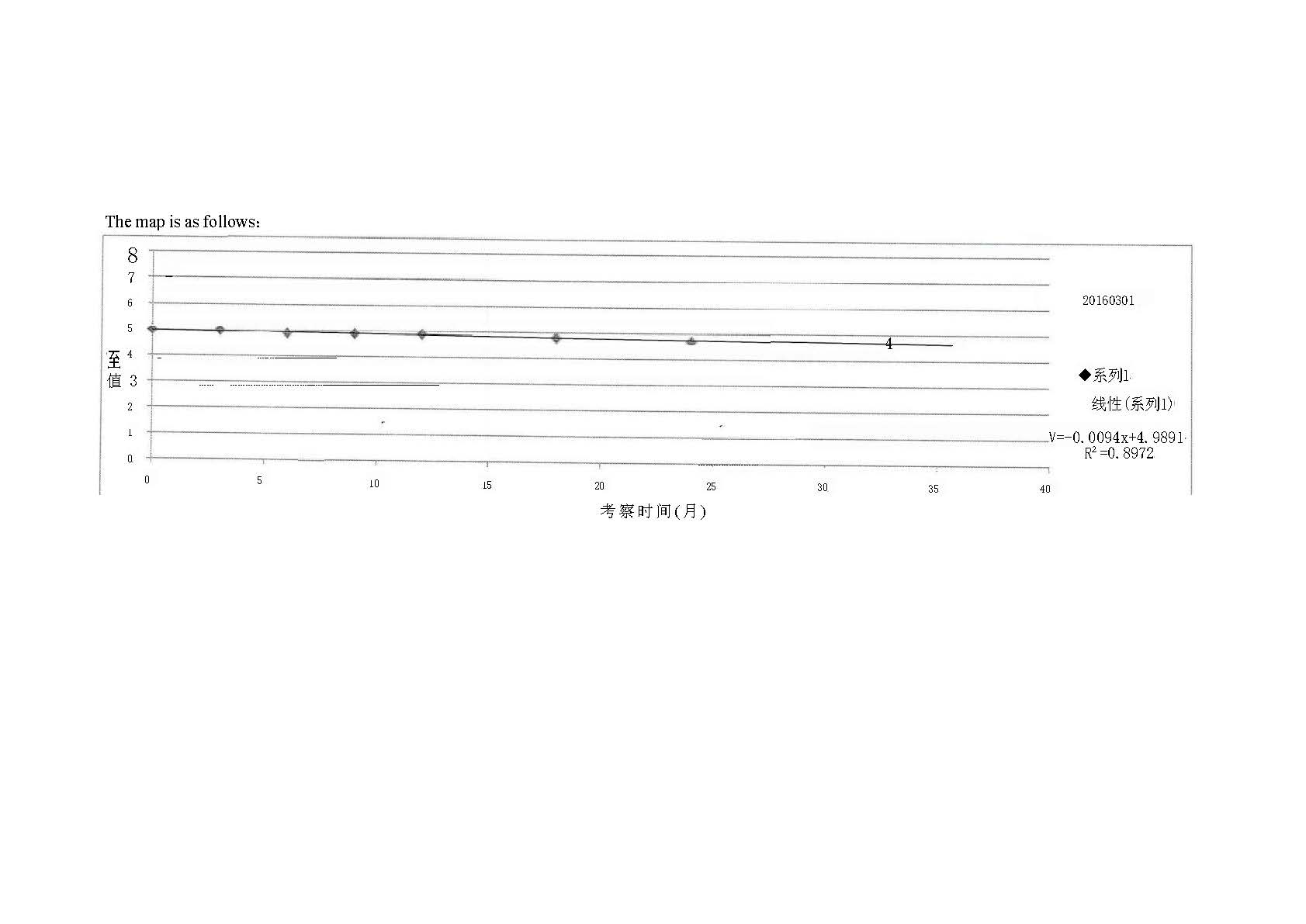


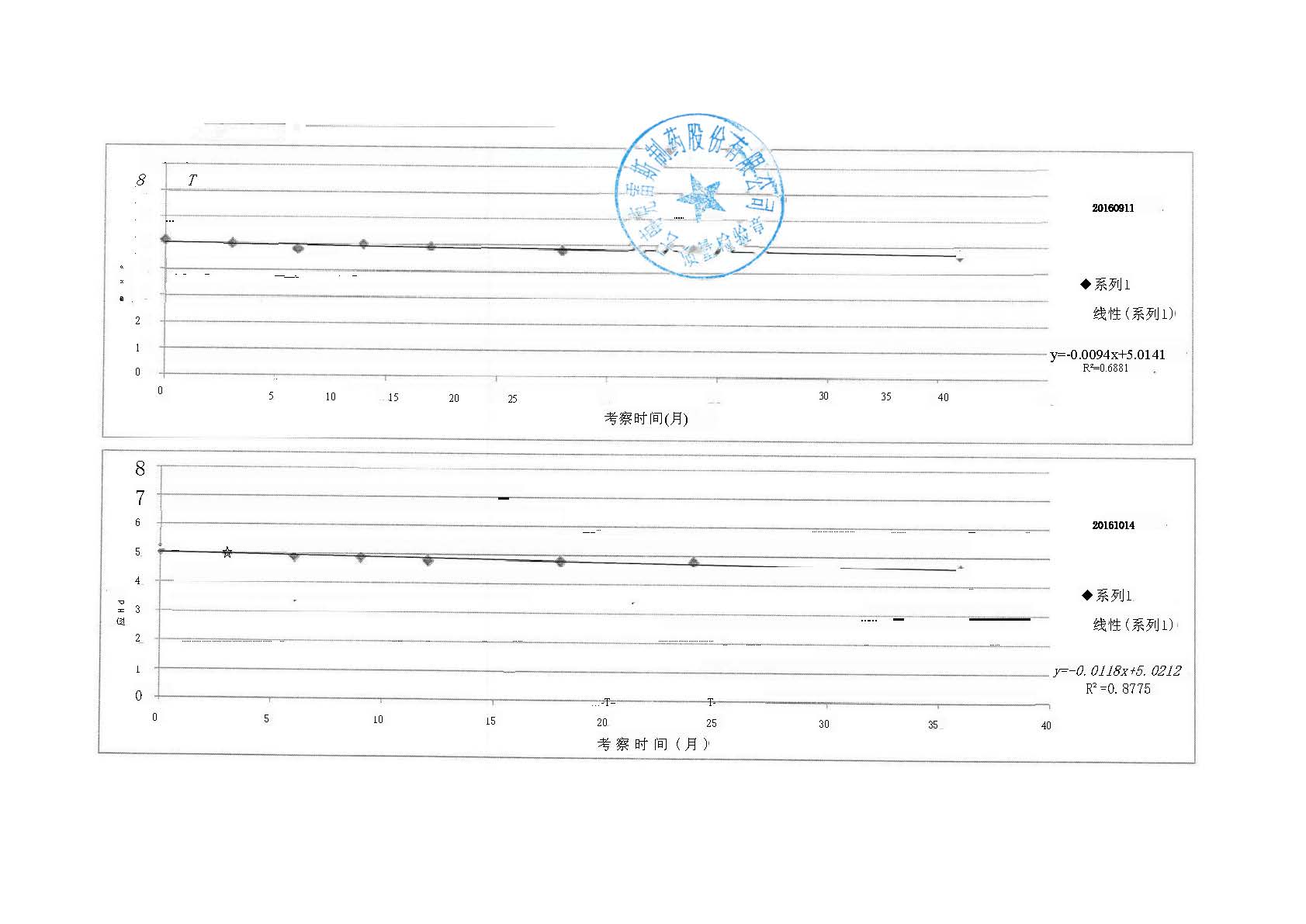


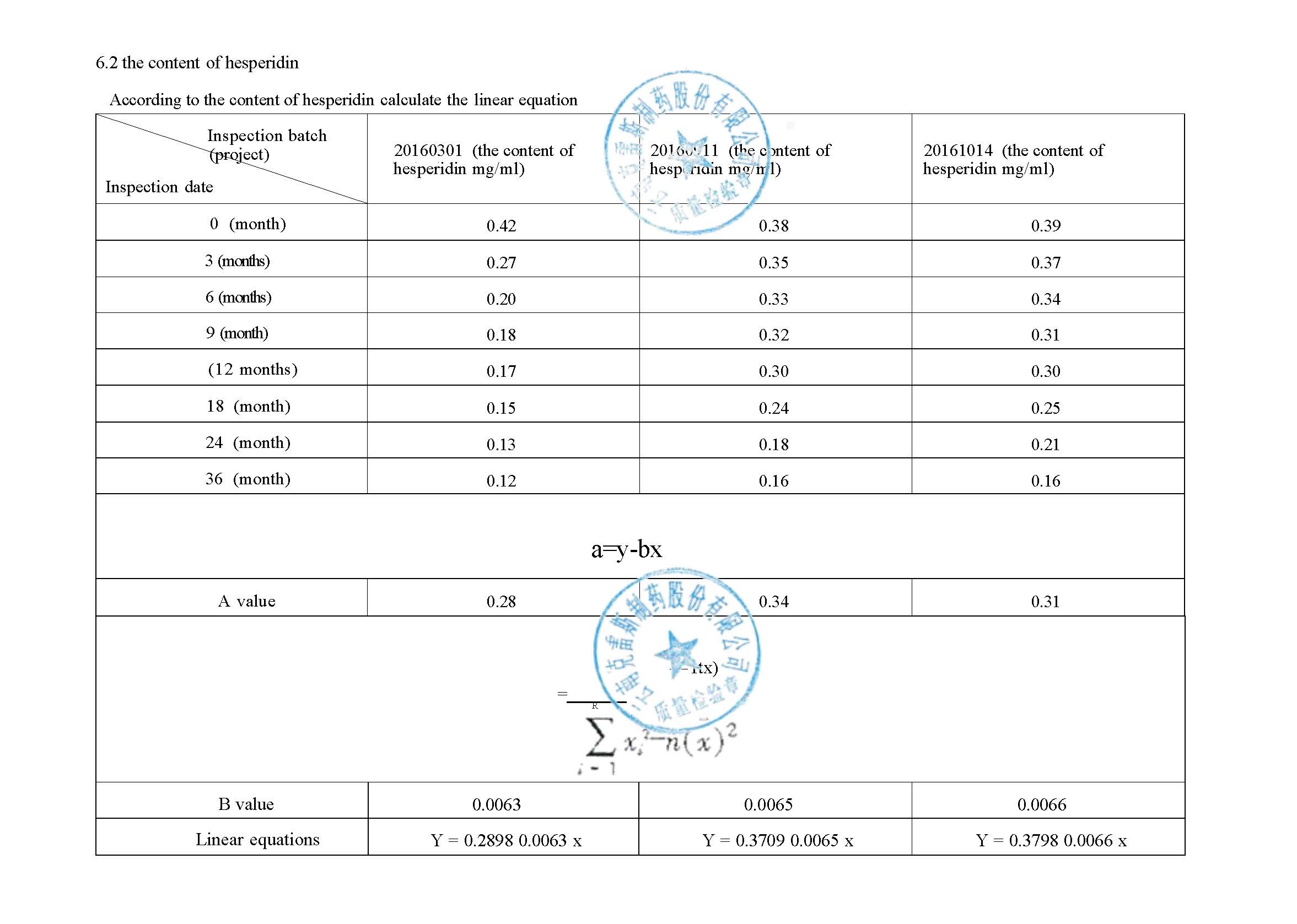


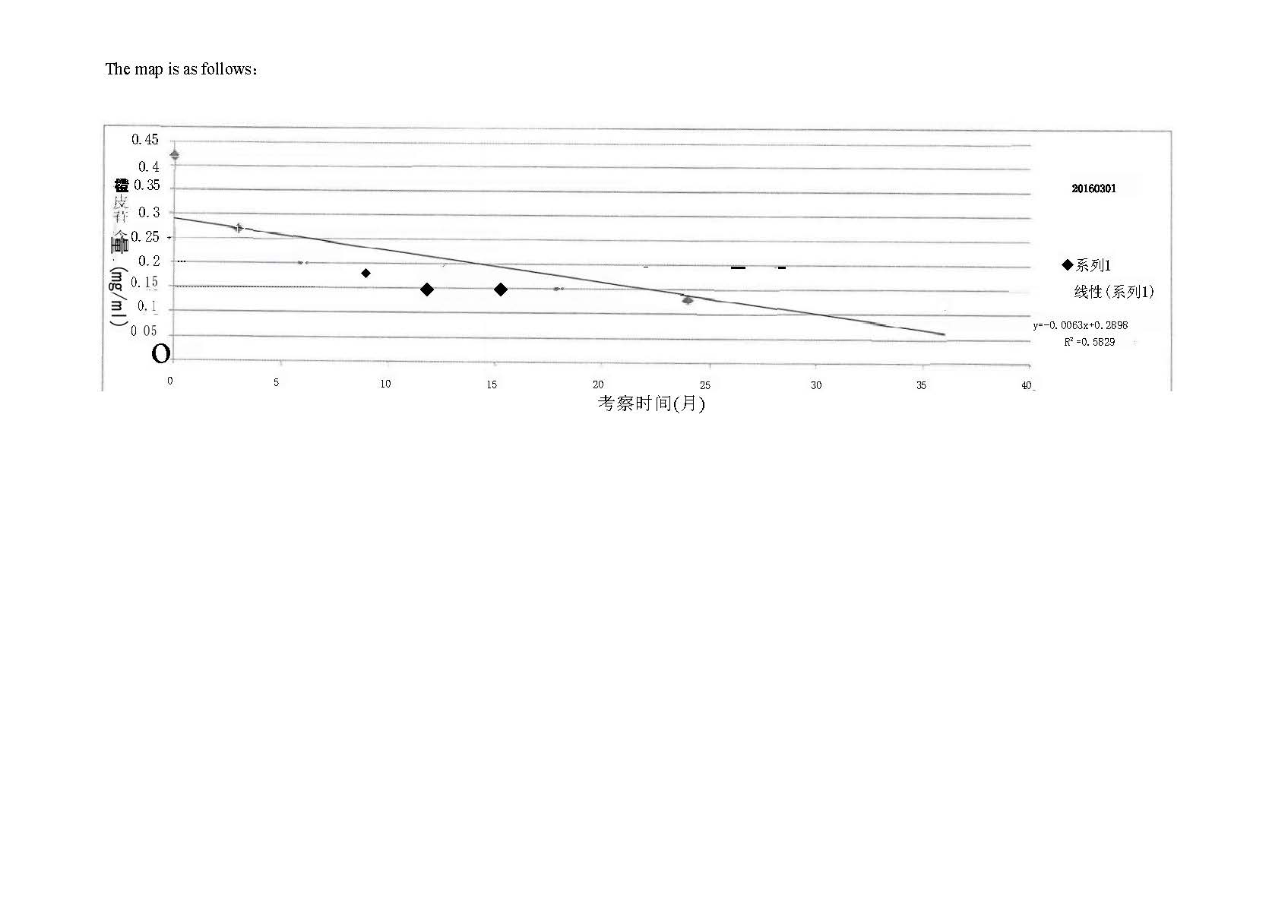


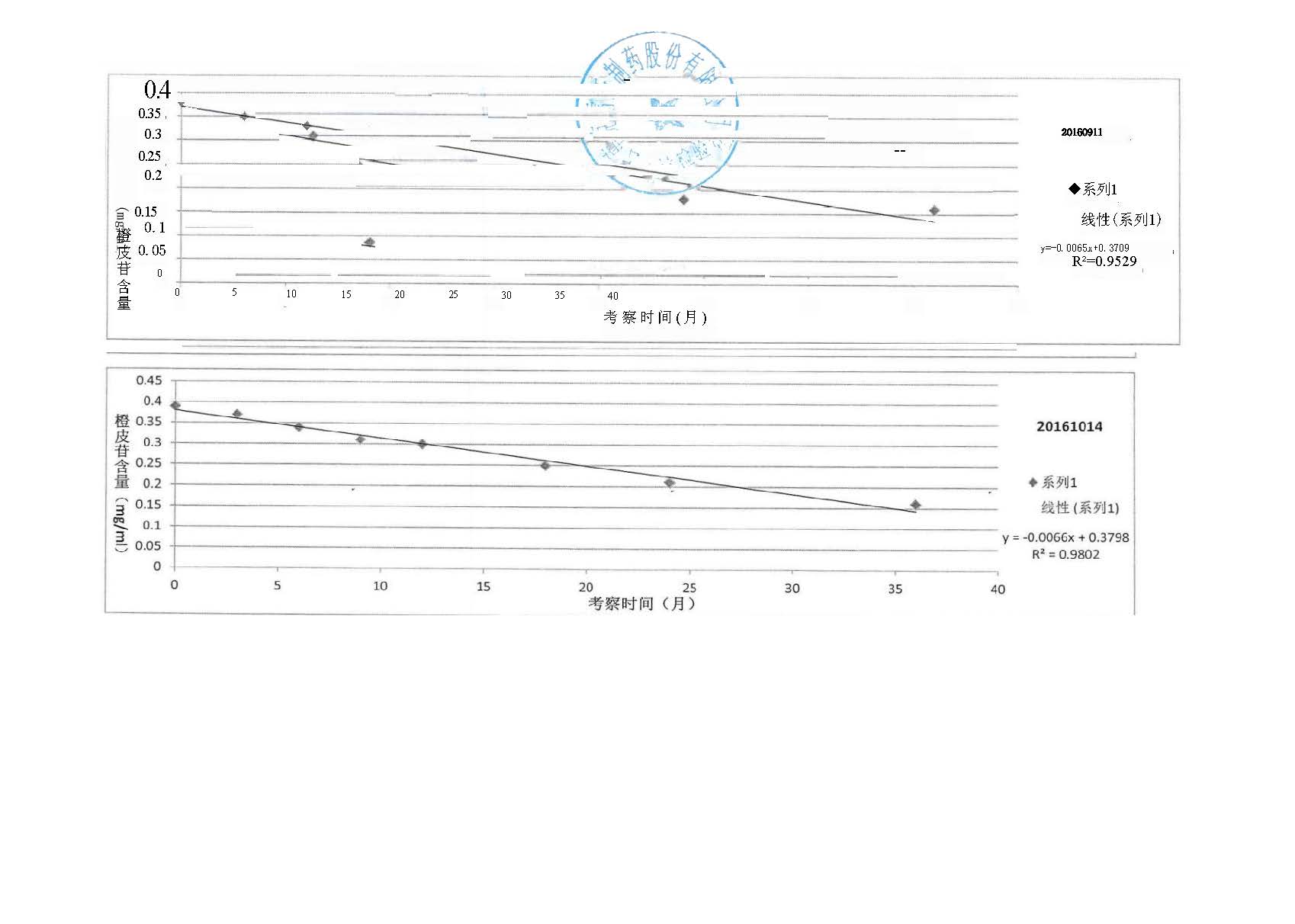


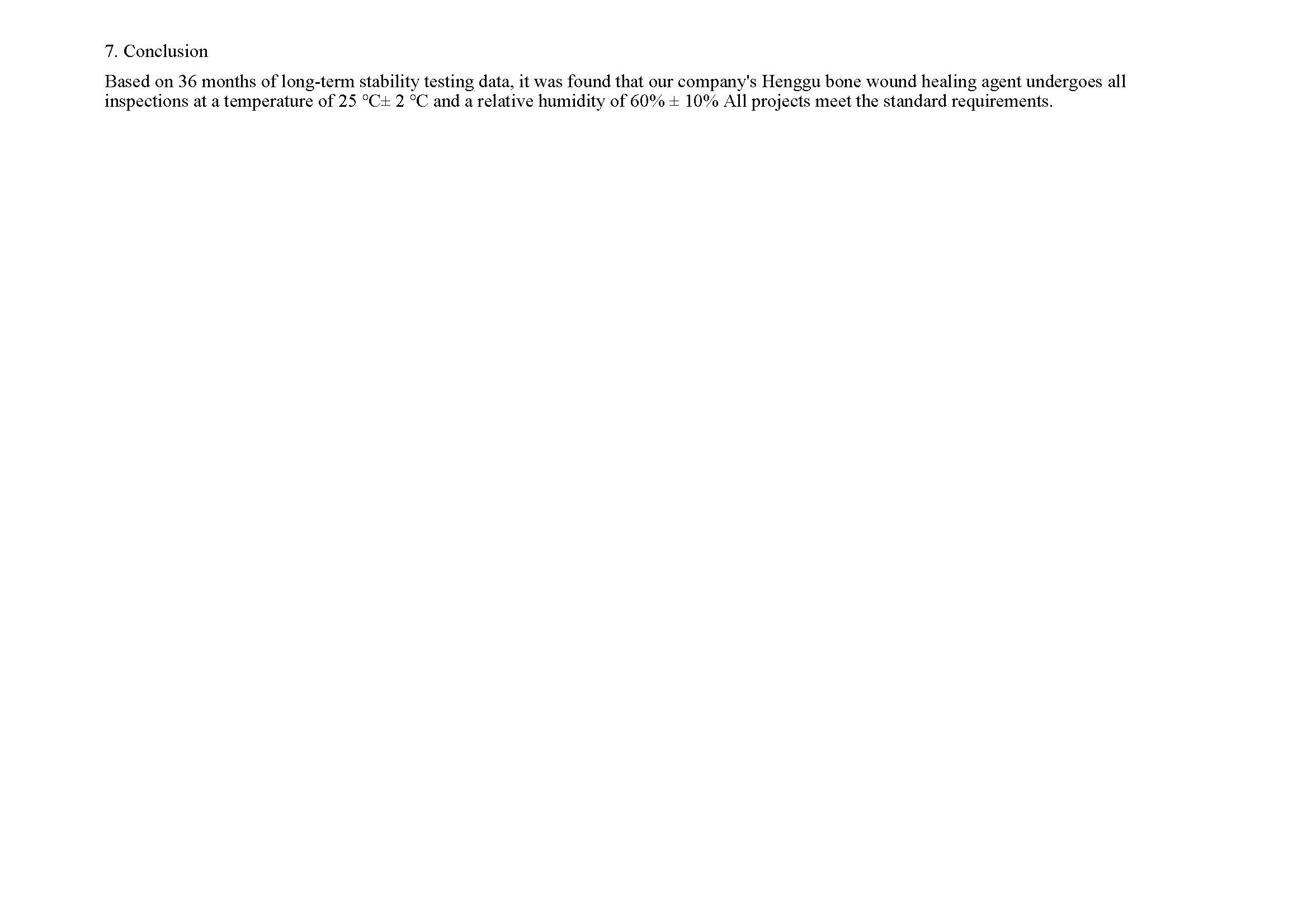


# Supplementary Material 15 Comparison of VAS scores between two groups at different time points(before Propensity Score Matching)

| Variable | Total  (n = 501) | Osteoking Group  (n = 428) | NSAIDs Group  (n = 73) | Statistic | P |
| --- | --- | --- | --- | --- | --- |
| baseline | 6.00 (5.00, 6.00) | 6.00 (5.00, 6.00) | 6.00 (5.00, 7.00) | Z=-4.05 | <0.001 |
| 2 weeks | 4.00 (4.00, 5.00) | 4.00 (3.00, 5.00) | 5.00 (4.00, 6.00) | Z=-5.37 | <0.001 |
| 4 weeks | 3.00 (2.00, 4.00) | 3.00 (2.00, 4.00) | 4.00 (4.00, 5.00) | Z=-6.13 | <0.001 |
| 8 weeks | 2.00 (1.00, 3.00) | 2.00 (1.00, 3.00) | 3.00 (2.00, 4.00) | Z=-5.36 | <0.001 |
